# Comparing intervention strategies for reducing Clostridioides difficile transmission in acute healthcare settings: an agent-based modeling study

**DOI:** 10.1186/s12879-020-05501-w

**Published:** 2020-10-28

**Authors:** Brittany Stephenson, Cristina Lanzas, Suzanne Lenhart, Eduardo Ponce, Jason Bintz, Erik R. Dubberke, Judy Day

**Affiliations:** 1 Department of Engineering, Computing, and Mathematical Sciences, Lewis University, 1 University Parkway, Romeoville, 60446 IL USA; 2 Department of Population Health and Pathobiology, North Carolina State University, 1052 William Moore Drive, Raleigh, 27606 NC USA; 3 Department of Mathematics, University of Tennessee, 1403 Circle Drive, Knoxville, 37996 TN USA; 4 Department of Electrical Engineering and Computer Science, University of Tennessee, 1520 Middle Drive, Knoxville, 37996 TN USA; 5 School of Arts and Sciences, Johnson University, Knoxville, 37998 TN USA; 6 Division of Infectious Disease, Washington University School of Medicine, 660 S Euclid Ave, St. Louis, 63110 MO USA

**Keywords:** *Clostridioides difficile*, Environmental transmission, Agent-based model, Stochastic model, Healthcare

## Abstract

**Background:**

*Clostridioides difficile* infection (CDI) is one of the most common healthcare infections. Common strategies aiming at controlling CDI include antibiotic stewardship, environmental decontamination, and improved hand hygiene and contact precautions. Mathematical models provide a framework to evaluate control strategies. Our objective is to evaluate the effectiveness of control strategies in decreasing *C. difficile* colonization and infection using an agent-based model in an acute healthcare setting.

**Methods:**

We developed an agent-based model that simulates the transmission of *C. difficile* in medical wards. This model explicitly incorporates healthcare workers (HCWs) as vectors of transmission, tracks individual patient antibiotic histories, incorporates varying risk levels of antibiotics with respect to CDI susceptibility, and tracks contamination levels of ward rooms by *C. difficile*. Interventions include two forms of antimicrobial stewardship, increased environmental decontamination through room cleaning, improved HCW compliance, and a preliminary assessment of vaccination.

**Results:**

Increased HCW compliance with CDI patients was ranked as the most effective intervention in decreasing colonizations, with reductions up to 56%. Antibiotic stewardship practices were highly ranked after contact precaution compliance. Vaccination and reduction of high-risk antibiotics were the most effective intervention in decreasing CDI. Vaccination reduced CDI cases to up to 90%, and the reduction of high-risk antibiotics decreased CDI cases up to 23%.

**Conclusions:**

Overall, interventions that decrease patient susceptibility to colonization by *C. difficile*, such as antibiotic stewardship, were the most effective interventions in reducing both colonizations and CDI cases.

## Background

*Clostridioides difficile*–an anaerobic, gram-positive, endospore-forming bacterium–is the leading cause of infectious diarrhea in United States hospitals and one of the most common healthcare-associated infections [1]. *C. difficile* colonizes the large intestine and can cause severe diarrhea and colitis [2]. In severe cases, *C. dfficile* also causes colonic perforation and death [2]. In 2019, the Centers for Disease Control and Prevention (CDC) classified *C. difficile* with the highest threat level of urgent [3]. *C. difficile* causes approximately 223,900 infections in hospitalized patients and 12,800 associated deaths in the U.S. per year [4]. The cost associated with *C. difficile* infection (CDI) in U.S. acute-care facilities alone has been estimated to be as much as <DOLLAR/>4.8 billion annually [5]. Because CDI is one of the most common nosocomial infections among patients in healthcare facilities, there is a critical need to better identify primary sources of transmission and optimal methods for prevention.

Controlling CDI is complicated because patient susceptibility to CDI is influenced by many factors, including antibiotic exposures. In addition, there are multiple sources of transmission; in hospitals, less than 35% of symptomatic cases are traced back to previous symptomatic cases [6, 7]. Other sources of transmission are asymptomatic carriers and the hospital environment. Colonized patients–both symptomatic and asymptomatic–shed *C. difficile* spores in the stool. Skin and environments near patients become contaminated with *C. difficile* spores [8]. *C. difficile* has been found on beds, sinks, toilets, walls, rails, call buttons, and stretchers [9]. The survival of *C. difficile* spores on hospital surfaces makes healthcare workers (HCWs) important vectors of transmission, particularly if they exhibit poor contact precaution practices [9].

Antibiotic use is the primary risk factor for CDI since antibiotics disrupt the normal gut microbiota [10], allowing *C. difficile* to colonize and proliferate [11]. Certain antibiotics may make individuals more susceptible to colonization by *C. difficile* than others. This depends on the spectrum, duration, and number of antibiotics received [12–14]. Prolonged duration of antibiotic use and number of prescriptions are both linked to increased risk for CDI [12]. Broad-spectrum antibiotics work against a broad range of bacteria, which results in more significant gut microbiota disturbance and, thereby, an increased risk for *C. difficile* colonization [14].

Common strategies aimed at controlling CDI include antibiotic stewardship, isolation of patients with CDI, environmental decontamination of rooms with bleach, and improved HCW hand-hygiene and contact protocol [15, 16]. Antibiotic stewardship may involve an overall reduction in the number of antibiotics prescribed and/or a reduction targeted specifically at the proportion of antibiotics prescribed that are associated with a high risk for CDI, such as fluoroquinolones, clindamycin, and cephalosporins [17–19]. Current hospital practice calls for the identification and subsequent isolation of patients with CDI so that proper contact precautions can be implemented to decrease the chances of pathogen spread [15, 16]. More purposeful cleaning with a sporicidal product is an additional strategy aimed at reducing the pathogen level in healthcare settings [20]. HCWs’ hands can become contaminated after touching CDI patients or surfaces with *C. difficile* spores [21, 22], and studies have shown that adherence to best hand-hygiene and contact protocol practices have been difficult to maintain [23, 24]. Because the spread of nosocomial pathogens has been linked to poor hand-hygiene practices [9], improved adherence of HCWs to proper hand-washing and contact protocols is also an important control measure. Overall, recommendations for controlling CDI have not changed over time. Vaccine trials have demonstrated hopeful results in clinical trials [25, 26]. Although many initial vaccines focus on toxin-related antigens and do not prevent colonization by *C. difficile*, vaccines may be an additional tool for reducing transmission in hospital settings by decreasing number of CDI patients.

Interventions are often combined; however, there are few clinical studies evaluating the effectiveness of combined interventions [27]. Computational models have been used to quantify the relative impact of single interventions on the spread of *C. difficile* and to determine the optimal combination of intervention strategies for reducing transmission [19, 24, 28]. Agent-based models (ABMs) allow us to define a system based on individual behaviors and interactions in order to observe emergent behaviors of the entire system [29]. ABMs also allow us to incorporate spatial heterogeneity, consider a variety of transmission pathways, and incorporate individual patient characteristics that are significant in determining transmission. Furthermore, ABMs inherently have stochastic components that can result in different outcomes from similar starting conditions.

Previous ABMs for *C. difficile* transmission differ on what elements of the transmission processes were included and subsequently what interventions the models were able to evaluate [19, 24, 28, 30, 31]. For example, the ABM created by Codella et al. [24] specifically considered patients, HCWs, and visitors as agents. Bintz et al. [19] developed an ABM that focused on evaluating the efficacy of various control measures targeting environmental contamination and antibiotic exposures in order to reduce colonization and infection incidence within the hospital. Previous ABMs did not simultaneously incorporate heterogeneity of antibiotics prescribed, individual patient antibiotic histories, and HCWs as vectors of transmission in their evaluation. In this study, we expand on the ideas formulated by Bintz et al. [19] in order to create an ABM of *C. difficile* transmission that incorporates specific patient histories, antibiotic histories, antibiotic risk levels, and explicitly incorporates HCWs as agents. By extending and modifying their model, we aim to evaluate the following control strategies:

1. Improved terminal cleaning and disinfection of environmental surfaces
2. Antibiotic usage restriction and stewardship
3. Improved HCW basic compliance
4. Improved HCW compliance with CDI patients
5. Vaccination.

## Methods

In this section, we give an overview of the structure and components in our ABM of nosocomial *C. diffcile* transmission. For all the specific details about model design and implementation in their entirety, see the Overview, Design Concepts, and Details (ODD) protocol provided as a supplemental file (Additional File 1), which also includes descriptions of the submodels. Our ODD protocol follows the standard formatting developed by Grimm, Railsback, and their collaborators [32].

### Model setting

We developed our ABM using NetLogo, a coding language and modeling environment primarily used for the creation of ABMs [33]. Our model is a modification and extension of the ABM originally created by Bintz et al. [19]. The model has two types of agents: patients and HCWs. The environmental patches represent ward rooms, and the model environment is a hospital consisting of six medical wards, each with 35 patient rooms. These are all single-patient rooms, so there can be at most 210 patients in the medical wards of the hospital at a time. For simplicity and to ensure we never have more patients than available rooms, we maintain a constant occupancy level. That is, the number of new patients admitted at a given time always equals the number of patients discharged at the previous time. For simplicity, the number of HCWs in the hospital is chosen to maintain a 3:1 ratio of patients to HCWs, and each time an HCW leaves the hospital, a new one arrives to maintain a constant total population of HCWs. In our model, HCWs may include physicians, nurses, assistants, cleaning staff, meal delivery persons, etc.

We note that shared equipment such as computers on wheels, vital signs machines, and glucometers can be a source of nosocomial pathogens in hospitals. Studies measuring the interaction between portable equipment and HCWs indicate that more than 50% of the contacts between portable equipment and patients are mediated by HCWs [34, 35]. For simplicity and computational efficiency, the model captures all the interactions between room components and patients through HCWs. As such, the model allows for HCWs to move from room to room but assumes that patients remain in their rooms.

We track behaviors and characteristics specific to each individual agent and each individual room. Patient interactions and characteristics are updated at every half-day time step, which mimics the time-scale of the ABM in [19], while HCW interactions and characteristics are updated at every 15-minute time step. Many of the values for the parameters used in the model were taken from [36], which was originally based on retrospective data collected from Barnes-Jewish Hospital in St. Louis, Missouri.

### Model components

For each ward room, we track its contamination level over time. The contamination level is unit-less and can be incremented or decreased based on pathogen transfer. The more surfaces in a room contaminated by *C. difficile* spores, the higher the room’s contamination level. Pathogen shedding of both symptomatic and asymptomatic patients will increase the contamination level of a room. However, we assume that the shedding of symptomatic patients contributes slightly more to room contamination than that of asymptomatic carriers since it has been shown that those with symptomatic CDI shed more *C. difficile* in their stool [16] and are more likely to be incontinent, which leads to more environmental contamination. We also note that although a more recent study has measured comparable levels of contagiousness of *C. difficile* carriers and CDI patients [37], our model is not particularly sensitive to the modestly different contamination levels used for asymptomatic versus symptomatic patients. Additionally, patients who are not colonized by *C. difficile* will have no effect on the contamination level of the room in which they are residing.

For all patients, we assign a length of stay in the hospital based on their disease status upon admission and on data for the lengths of stays of patients [19, 36]. We also track each patient’s time since admission, and once a preassigned length of stay is reached, the patient is discharged from the hospital.

All patients, regardless of their disease status with respect to *C. difficile*, have a probability of receiving an antibiotic (for treating illnesses not related to *C. difficile*) at each half-day time step. Because different types of antibiotics result in varying degrees of microbiota disturbance and, therefore, varying risks of colonization by *C. difficile* [12, 13], we group antibiotics into three risk levels with respect to *C. difficile*: low-, high-, and very high-risk [19]. The risk level of an antibiotic directly affects the time until restoration of a normal gut microbiota and a patient’s incubation period (the time between exposure to *C. difficile* and the onset of symptoms). In particular, higher risk antibiotics are associated with shorter incubation periods and longer periods until successful restoration of the gut microbiota [19]. The model tracks the number and associated risk level of antibiotics each patient receives. Additionally, the amount of time each patient spends on a particular antimicrobial therapy is tracked. Because of this, we are able to incorporate the impact of the antibiotic type, duration of treatment, and number of antibiotics on the risk of colonization and subsequent infection by *C. difficile*. A patient’s assigned length of stay is also updated based on the number and type of antibiotics he or she received while in the hospital.

Throughout a patient’s stay, we track the progression of his or her disease status, and we begin by noting the patient’s disease status at admission. There are four possible disease statuses of patients: resistant, susceptible, (asymptomatically) colonized, and diseased. Upon admission, a patient’s disease status is determined based on the admission proportions for each disease class. We use the same admission proportions here as used in [38], in which the proportions were based on hospital data with modifications made to the colonized admission proportion because of updated data given by Alasmari et al. [39].

**Fig. 1:**
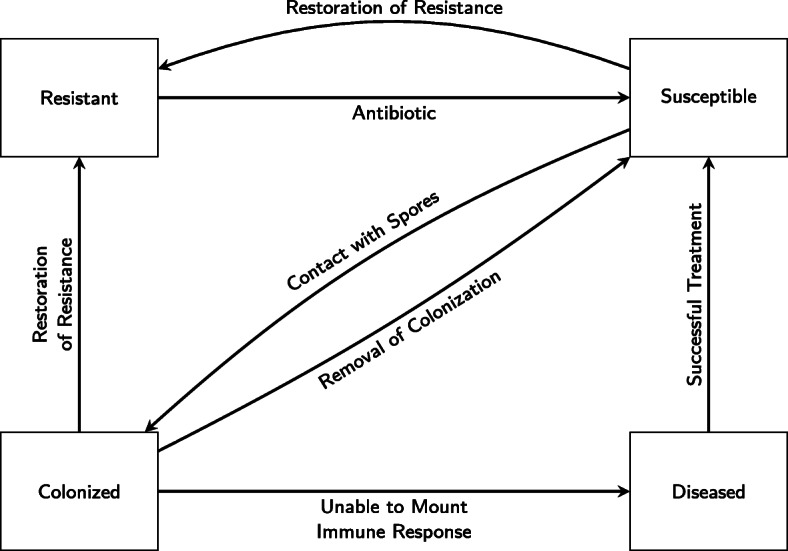
Summary of movement among disease statuses of patients in ABM

All possible transitions among disease states are represented in Fig. 1. Because antibiotic use is widely recognized as the most significant risk factor for colonization by *C. difficile* [2, 40], we assume a patient only becomes susceptible to colonization after beginning antimicrobial therapy [38]. Those who have not recently undergone antimicrobial therapy are considered resistant to colonization and will not be affected by exposure to *C. difficile* spores. Since, on average, a patient’s microbiota will return to normal after 30 days [36], our model allows susceptible patients to return to resistant if they are not exposed to *C. difficile* while susceptible or if they do not receive an additional antibiotic in those 30 days. However, the susceptible individuals who do come into contact with *C. difficile* spores have a chance of becoming colonized. Each individual susceptible patient has his or her own probability of becoming colonized that changes at each 15-min time step and depends on the risk level of the antibiotic prescribed and on the contamination level of his or her room.

Upon colonization, patients are randomly assigned as either lacking protective immunity or not, indicating whether or not they mount their own immune response against the toxins produced during colonization. In keeping with the values used by Bintz et al. [19], there is a 10% chance a colonized individual will be lacking protective immunity. This leads to approximately 1 symptomatic patient for every 9 asymptomatic patients. All colonized patients have a chance of receiving one or more additional antibiotics (for the treatment of illnesses not related to *C. difficile*). For those who are not lacking protective immunity and receive an additional antibiotic, it is possible they will clear their colonization but still have an altered gut microbiota. Therefore, this subset of colonized patients may return to susceptible. For colonized patients who are not lacking protective immunity and do not receive an additional antibiotic, they may clear their colonization and also have their gut microbiota return to normal. This subset of colonized patients will return to the resistant class. Finally, those colonized who are lacking protective immunity become clinically infected. If they receive an additional antibiotic prior to becoming diseased, this will shorten their incubation period and cause them to experience CDI symptoms more quickly than they would have otherwise. Our model includes screening of symptomatic patients for CDI with the turnaround time for the screening test and the sensitivity of the screening test also incorporated. Within a half-day time step or two half-day time steps, a symptomatic patient may be tested and receive the results. Additionally, the symptomatic patients who were unsuccessfully screened will be tested again in a subsequent time step. Those diseased patients who are successfully screened for CDI will be quarantined, and their symptoms will be treated with one of the typical antibiotics used to treat CDI. There is an 80% chance of successful treatment and resolution of symptoms that will allow diseased patients to return to the susceptible class [36].

Upon arrival to the hospital, each HCW is assigned a shift length. For simplicity, we consider either 8-hour or 12-hour shifts, with a 50% chance of each. We track each HCW’s time since beginning a shift, and once this time surpasses the total shift length assigned, that HCW leaves the hospital. We divide HCWs into two groups: Type 1 and Type 2. Type 1 HCWs are assumed to be completing more routine, less time-consuming tasks and, therefore, move from patient to patient every 15 min. In contrast, Type 2 HCWs spend more time with the patients they visit and only move from patient to patient every 45 min. Our model assumes that no HCW will visit a vacant room and that HCWs of the same type will never be in the same room simultaneously; however, a Type 1 and Type 2 HCW may visit the same patient at the same time. HCWs have individual contamination levels that represent the amount of *C. difficile* they are carrying. Like room contamination levels, these are unit-less and will be incremented and decreased based on pathogen transfer. We do not track *C. difficile* colonization or infection of HCWs and view them only as vehicles of pathogen spread from room to room. Therefore, we assume each HCW has a contamination level of zero upon entry into the hospital. Each time an HCW visits a patient, the chances of becoming contaminated by *C. difficile* depends on the type of task being performed on the patient and on the amount of contamination in the room. For this reason, we divide the types of tasks HCWs complete into three groups: low-, medium-, and high-risk. The risk associated with a particular task depends on the invasiveness of the task and the likelihood of coming into contact with a large number of surfaces in the room. For example, we consider a task such as giving a patient a scrub bath to put an HCW more at risk of becoming contaminated than giving medication to a patient. Although both types of HCWs can perform any level of task, we assume Type 1 HCWs have a greater chance of performing low-risk tasks while Type 2 HCWs have a greater chance of performing high-risk tasks.

### Model processes

Before beginning simulations, the model environment is first initialized. In this process, we populate the hospital with enough patients to meet the specified occupancy level. The disease status of these patients upon admission is based on the admission proportions: *a*_*r*_,*a*_*s*_,*a*_*c*_, and *a*_*d*_, whose values are given in Table 1. The room contamination levels are then initialized based on the disease status of the patient in the room. Rooms with resistant or susceptible patients will have a contamination level of zero. Because colonized and diseased patients will shed *C. difficile* spores, the contamination level of their rooms will be increased to reflect this. The exact process for determining the amount of increase is described in the ODD protocol (Additional File 1), where each submodel of the ABM is outlined. The hospital is next populated with HCWs, and the contamination level of all HCWs is initialized to zero. In this initialization process, HCWs are randomly assigned a length of time remaining on their shift, varying from 0 to 12 hours remaining before leaving the hospital. After the initialization process, all shifts will either be 8 or 12 hours long. We let the model run for a three-week time period before recording outputs to ensure the resulting outputs are not significantly dependent on the specified initial conditions.

**Table 1:** Global variable explanations and baseline values

Global variable	Description	Baseline value
occupancy	hospital occupancy level	0.85
*a*_*r*_	probability a patient is resistant upon admission	0.75
*a*_*s*_	probability a patient is susceptible upon admission	0.09
*a*_*c*_	probability a patient is colonized upon admission	0.15
*a*_*d*_	probability a patient is diseased upon admission	0.01
probability-lack-immunity	probability a colonized patient will not mount an immune response	0.1
*p*_*rrmin*_	minimum probability of regaining resistance	0.2
probability-antibiotic	half-daily probability of a patient beginning an antibiotic treatment	0.27
prob-low-risk	probability of a prescribed antibiotic being low-risk with respect to CDI	0.4
prob-high-risk	probability of a prescribed antibiotic being high-risk with respect to CDI	0.26
prob-vhigh-risk	probability of a prescribed antibiotic being very high-risk with respect to CDI	0.34
$p_{l}^{h}$	probability of becoming colonized if treated with low-risk antibiotic in a highly contaminated room	1/30
prob-suff-clean	probability of effective room cleaning	0.5
sensitivity	sensitivity of the CDI screening test	0.91
turnaround	turnaround time (half-days) of the CDI screening test	2
prob-succ-treat	probability of successful treatment of CDI	0.8
HCW-compliance-CDI-patients	probability of an HCW following proper contact precautions when visiting a quarantined patient with CDI	0.6
HCW-basic-compliance	probability of an HCW complying with contact precautions after visiting a non-quarantined patient	0.45
clean-reduction	proportion by which the contamination level of a room is reduced after effective cleaning	0.5
HCW-transfer-percent	proportion of an HCW’s carrier level that is transferred to a room upon successful transfer	0.9
room-transfer-percent	proportion of a room’s contamination level that is transferred to an HCW upon successful transfer	0.1
contam-level-low	maximum contamination level of a low-contamination room	0.4
contam-level-med	maximum contamination level of a medium-contamination room	0.8

**Fig. 2:**
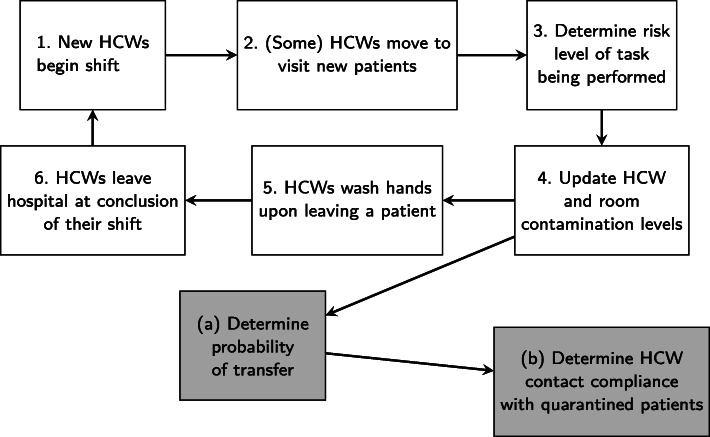
Summary of ABM processes that are run at each 15-minute time step

After initialization, the model executes the processes outlined in Fig. 2 at every 15-min time step. To update the contamination levels of rooms and HCWs, we first determine the probability of pathogen transfer occurring. When HCWs visit rooms, they have a chance of picking up *C. difficile* spores, which would add to the existing contamination on their hands, and they also have a chance of transferring *C. difficile* spores already on their hands to the room. The probability of an HCW picking up pathogen when visiting a room depends on the amount of contamination in the room and the risk level of the task being performed. Similarly, the probability of an HCW transferring pathogen from his or her hands to a room surface depends on the contamination level of the HCW and on the risk level of the task being performed. We refer to the chance of HCWs picking up pathogen from the room as *prob-room-transfer* and refer to the chance of HCWs transferring some of their existing contamination to the room as *prob-HCW-transfer*. To calculate these probabilities at each 15-min time step for each HCW and room, we use three transfer functions, one for each task risk level. When determining *prob-room-transfer*, we consider these transfer functions to be functions of the room contamination level, and when determining *prob-HCW-transfer*, we consider them to be functions of the HCW contamination level. For more details and to see the specific functions used, refer to the ODD protocol (Additional File 1). Based on these probabilities, the model then determines whether or not transfer will occur between a room and an HCW.

Studies have shown that HCWs are more likely to adhere to contact protocol when visiting patients in isolation [41]. We refer to the likelihood of HCWs following compliance protocol when visiting quarantined patients as *HCW-compliance-CDI-patients* and set its baseline value to 0.6 [42]. If HCWs properly comply (based on the 60% chance), then there is a 0% chance of pathogen transfer between that room and the HCW; if they do not comply, there is a 100% chance of some transfer occurring. We keep in mind that this may be a strong assumption as we are assessing the effectiveness of interventions.

After determining whether transfer will occur for each HCW-room combination, the model next updates HCW and room contamination levels to reflect the transfer. Contamination levels are unit-less numbers that are either increased or decreased depending on the result of pathogen transfer. In particular, if an HCW picks up pathogen from a room, we decrease the room contamination level by 10% and increase the HCW contamination level by that same amount to represent the transfer. Similarly, if an HCW transfers pathogen to a room, we decrease the HCW contamination level by 90% and increase the room contamination level by the same amount. These percentages were chosen to reflect the fact that ward rooms contain many surfaces, so one HCW is likely to only pick up a small percentage of the total contamination in the room during one visit. In contrast, we are only tracking the contamination of HCWs on their hands, so they are likely to transfer a vast majority of their total contamination to the room if it is determined that a transfer will occur.

HCWs may decrease their contamination levels by following proper compliance protocol. Note that the ABM does not simulate any specific practices being implemented to achieve such a decrease in contamination, but only their overall efficacy at reducing contamination. In particular, there is a 45% chance of an HCW reducing his or her contamination level after a visit with a patient. This value was obtained by averaging the adherence percentage after patient contact of nurses and physicians given by Rubin et al. [28]. We refer to this compliance as *HCW-basic-compliance* and set its baseline value to 0.45 (Table 1).

**Fig. 3:**
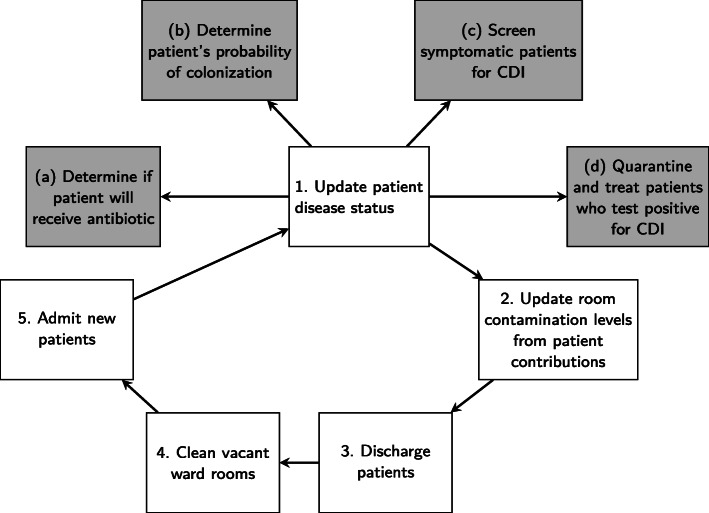
Summary of ABM processes that are run at each half-day time step

In addition to the processes run at each 15-min time step, the model runs the processes in Fig. 3 at each half-day time step. At each half-day time step, all patients have a 27% chance of receiving an antibiotic. This number was chosen by Bintz et al. [19] so that the output for the total number of antibiotic treatments per patient matched the data from Barnes-Jewish Hospital. Whether or not a patient receives an antibiotic will affect his or her disease status. When the model updates each patient’s disease status, it first determines if that patient will receive an antibiotic and then determines the risk level of the antibiotic given. In this step, the model also updates the number of antibiotics each patient receives. The changes to disease status are updated based on the transitions previously described and illustrated in Fig. 1.

**Fig. 4:**
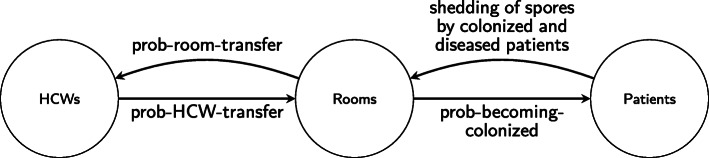
Summary of modes of *C. difficile* transmission included in the ABM, where *prob-room-transfer* refers to the probability of an HCW picking up *C. difficile* spores from a room, *prob-HCW-transfer* refers to the probability of an HCW contaminating room surface(s) with spores, and *prob-becoming-colonized* represents the probability of a patient becoming colonized based on the contamination level of the room and on the risk level of the antibiotic received

After patient disease statuses are updated, the room contamination levels are updated based on contributions from CDI and asymptomatically colonized patients. A summary of all possible *C. difficile* transfer routes is represented in Fig. 4. Note that our model does not explicitly include pathogen transfer directly from HCW to patient or vice versa; rather, we model the transfer between HCWs and rooms and between patients and rooms.

Once the disease status of patients and room contamination levels are updated, patients whose time since entering the hospital exceeds their length of stay assigned at admission are discharged. After this, the model admits new patients to replace those who were just discharged. This admission of patients is run in the same way patients were admitted during the initialization process. When new patients are admitted, we assign them a disease status and initialize all other patient characteristics that we are tracking. A complete list of these patient characteristics is given in the ODD protocol (Additional File 1) along with a detailed description of the admission of patients process run by the model.

### CDI prevention interventions

Our goal is to compare the impact of various prevention interventions, and combinations of prevention interventions, on the transmission of and subsequent infection by *C. difficile*. We consider the following intervention strategies:

1. Antimicrobial stewardship (in two forms)
2. Increased HCW adherence to compliance protocol (with non-quarantined patients and specifically with quarantined patients)
3. Improved environmental decontamination
4. Vaccination (a preliminary assessment).

We consider antimicrobial stewardship in two forms (using the same techniques described in [19]): (1) an overall reduction in the number of antibiotics prescribed and/or (2) a reduction targeted at the proportions of high-risk and very high-risk antibiotics prescribed. To implement an overall reduction in the number of antibiotics prescribed, we reduce the half-daily probability of a patient receiving an antibiotic by a certain proportion. At baseline, this reduction is assumed to be 0% so that there is a 27% chance of patients receiving an antibiotic each half-day, as described by the global variable *probability-antibiotic* listed in Table 1. The intervention scenarios considered include a reduction of this probability by 10% and by 20%, resulting in a 24.3% and a 21.6% chance, respectively, of patients receiving an antibiotic each half-day. For easy reference, these values are given in Table 2.

**Table 2:** Antimicrobial stewardship strategy: reduction in the overall number of antibiotics prescribed

	Probability of receiving antibiotic
baseline	0.27
10% reduction	0.243
20% reduction	0.216

To implement the second form of antimicrobial stewardship, we alter the probabilities of the antibiotic prescribed being low risk, high risk, or very high risk with respect to CDI. The scenarios considered here are the same as those used by Bintz et al. [19] and are listed in Table 3. In the baseline scenario, we set the proportion of low-risk antibiotics prescribed to be 0.4, the proportion of high-risk to be 0.26, and the proportion of very high-risk to be 0.34. We will refer to this as targeted antibiotic reduction scenario 1. The second targeted antibiotic reduction scenario considered involves reducing the probability of very high-risk antibiotics being prescribed by half, and as a result, increasing the probability of high-risk antibiotics being prescribed by that same amount. The third and final targeted antibiotic reduction scenario considered involves replacing both half of the proportion of very high-risk antibiotics prescribed with high-risk antibiotics and half of the high-risk antibiotics prescribed with low-risk antibiotics.

**Table 3:** Antimicrobial stewardship strategy: reduction in the proportions of antibiotics prescribed according to risk level with respect to CDI

Targeted antibiotic reduction scenario	1	2	3
proportion of low-risk	0.4	0.4	0.53
proportion of high-risk	0.26	0.43	0.3
proportion of very high-risk	0.34	0.17	0.17

The next intervention strategy considered involves the increased adherence of HCWs to contact precaution protocols. We consider both improved HCW compliance with each patient visit and improved HCW compliance specifically while visiting quarantined patients. The baseline value for HCW compliance after completing a routine visit with a patient is 0.45, as previously mentioned to be taken from data given in [28], and is referred to as *HCW-basic-compliance*. To assess the impact of improved HCW adherence on transmission and infection, we consider values greater than 0.45, including 0.65, 0.75, 0.85, and 1. Although a compliance of 100% is not the most likely scenario, this extreme case allows us to determine the impact of this particular control intervention. In addition to this more basic HCW compliance, we also consider an increase in compliance when visiting quarantined patients with CDI, referred to as *HCW-compliance-CDI-patients*. The baseline value for this compliance is 0.6, and we increase it by various amounts up to, and including, 1 to assess the impact of this control strategy. (Details given in Additional File 1.)

The third intervention strategy considers improved environmental decontamination. In this paper, we only present the results for terminal cleaning of rooms after patients are discharged rather than routine daily cleaning of all rooms. Different types of cleaning and disinfection strategies will have varying impacts on the removal of *C. difficile* spores in the environment. Thus, we incorporate a probability of sufficient cleaning into our model with a baseline value of 0.5 [19] that represents the effectiveness of routine terminal cleaning. This means there is a 50% chance that the cleaning will reduce the contamination level in the room by some amount. An increased value of this probability indicates the implementation of a more stringent and effective cleaning strategy that targets *C. difficile*. Additionally, we increase this probability of sufficient cleaning when simulating the cleaning of a room for a quarantined patient.

The *C. difficile* vaccines currently being tested are toxoid vaccines that will not protect against colonization by *C. difficile*. An effective version of this vaccine would result in a decrease in the proportion of patients lacking protective immunity. We perform a preliminary assessment of vaccination by making the strong assumption that a vaccination program has been successfully implemented for a period of time such that it has already resulted in an overall reduction in the percentage of patients lacking protective immunity. To simulate this, we decrease the baseline value for the probability of a patient lacking protective immunity from 10%, used by Bintz et al. [19], down to values such as 5% or 1%. Note that this is a preliminary assessment of vaccination because it relies on the major assumptions that all patients at risk will be vaccinated and will be vaccinated effectively leading to a reduction in the proportion of patients who experience clinical symptoms of CDI.

To assess the impact of the interventions, we examine the resulting number of nosocomial colonizations and nosocomial infections over a year’s time period under each particular control strategy. Because simulations result in varying numbers of total patient admissions per year, we normalize all of the outputs to 10,000 admitted patients per year for comparison. Because of the stochasticity embedded in ABMs, to best assess the impact of the control intervention strategies, we ran our model for 100 iterations over a one-year simulated time period with each combination of parameter values (representing different control strategies) using the technique outlined by Ponce et al. in [43].

## Results

### Baseline results

The entire list of baseline parameter values is given in Table 1. The median number of nosocomial colonizations per year at baseline normalized to 10,000 admissions was 2,157, and the median number of nosocomial infections per year normalized to 10,000 was 111. We adjusted select parameters from Bintz et al. [19] purposely so that our numbers of nosocomial colonizations matched updated data indicating that nosocomial colonization incidence affects 20% of admitted patients [40, 44] (details described in Additional File 1). Throughout the results section, we report median numbers only, but we note that the mean numbers were never significantly different from the corresponding medians. Thus, we did not have significant outlier cases.

In the baseline scenario, the majority of patients who become colonized were admitted as resistant; thus they became both susceptible and colonized during the hospitalization (approximately 19.64% of the total number of admitted resistant patients). The high number of resistant patients becoming colonized is a reflection of the fact that patients have a 75% chance of being resistant upon admission, so the number of resistant patients hospitalized is much higher than that of any other disease state. The percentage of admitted susceptible patients who become colonized was 32.87%. The largest percentage of admitted patients who will become clinically infected are those who were colonized upon admission (5.56%). Less than 1% of resistant and susceptible became diseased.

### Single prevention interventions

The baseline values of all parameters to be varied and the median number of nosocomial colonizations and infections for the baseline scenario are specified in Table 4. Table 5 gives the resulting percentage decrease in median nosocomial colonizations and infections for each of the individual control interventions.

**Table 4:** Baseline values of the variables that were varied to simulate control scenarios and median numbers of nosocomial colonizations and infections for a 1-year period normalized to 10,000 admissions

Variable	Baseline value
probability-antibiotic	0.27
targeted-antib-reduction-scenario	1
prob-suff-clean	0.5
HCW-basic-compliance	0.45
HCW-compliance-CDI-patients	0.6
probability-lack-immunity	0.1
Median nosocomial colonizations	2157
Median nosocomial infections	111

**Table 5:** Percentage reduction in the baseline median number of nosocomial colonizations and infections (given in Table 4) for each of the individual control scenarios, where the specific distributions of antibiotic-risk-level probabilities for each targeted antibiotic reduction scenario are given in Table 3

Preventive strategy	Decrease from median baseline nosocomial colonizations	Decrease from median baseline nosocomial infections
**Reduction in overall antibiotics**		
probability-antibiotic =0.243 (by 10%)	10.04%	5.21%
probability-antibiotic =0.216 (by 20%)	19.33%	8.45%
**Reduction in vhigh- and high-risk antibiotics**		
targeted-antib-reduction-scenario =2	7.93%	14.90%
targeted-antib-reduction-scenario =3	17.45%	23.21%
**Improved terminal cleaning**		
prob-suff-clean =0.8	2.26%	2.10%
prob-suff-clean =1	4.13%	2.76%
**Increased HCW compliance**		
HCW-basic-compliance =0.75	8.73%	5.47%
HCW-basic-compliance =1	14.73%	5.87%
**Increased HCW compliance with CDI patients**		
HCW-compliance-CDI-patients =0.8	7.06%	1.99%
HCW-compliance-CDI-patients =1	55.97%	15.69%
**Vaccination**		
probability-lack-immunity =0.05	0.70%	50.29%
probability-lack-immunity =0.01	2.46%	90.22%

Increasing HCW compliance with quarantined patients has the largest impact on reducing nosocomial colonizations while vaccination is the most effective method for reducing nosocomial infections (Table 5). When comparing the two antimicrobial stewardship strategies, we note that reducing the proportions of high-risk and very high-risk antibiotics has more of an impact on decreasing the nosocomial infections than reducing the half-daily probability of receiving an antibiotic has on reducing nosocomial infections. In fact, reducing the proportions of very high-risk and high-risk antibiotics is the second most effective strategy for reducing nosocomial infections (second to vaccination). Improved terminal cleaning does not have a notable impact on colonizations or infections even if 100% effective terminal cleaning is maintained. Increased HCW basic compliance has a comparable impact on nosocomial colonizations to that of antimicrobial stewardship (both forms); however, improving the basic HCW compliance does not have much impact on reducing nosocomial infections, especially when compared to that of targeted reduction in high-risk and very high-risk antibiotics.

If HCWs achieve 100% compliance with quarantined patients, there is an extremely noticeable decrease in nosocomial colonizations (55.97%). Such a vast decrease is likely due to our model structure: if HCWs are compliant with quarantined patients, there is a 0% chance of pathogen transfer from HCW to room, or vice versa. This assumes that when fully compliant, HCWs will strictly follow contact protocol so that there is no chance of exposure or transfer, which is more likely to happen when HCWs know a patient is isolated due to symptomatic CDI. This control strategy also results in a sizable reduction of nosocomial infections.

Finally, since vaccination does not prevent colonization, it has a minimal impact on the nosocomial colonizations, but a large impact on the nosocomial infections. In fact, there is not one individual strategy that is best at reducing both nosocomial colonizations and nosocomial infections simultaneously.

### Combination strategies

We begin this section by considering various combinations of antimicrobial stewardship and improved terminal cleaning. The combinations of antimicrobial stewardship and terminal cleaning considered were taken from [19] and are numbered by scenario in Table 6; they include all three levels of reduction in overall antibiotics that were individually implemented (baseline, 10% reduction, and 20% reduction), all three targeted high-risk and very high-risk antibiotic reduction combinations (targeted antibiotic reduction scenario 1, 2, and 3 given in Table 3), and all three probabilities of sufficient cleaning considered individually (0,2, 0.5, and 0.8). Note that the baseline combination scenario is indicated by Scenario 2 in Table 6. As the results of implementing strategies individually suggested, nosocomial colonizations are not as greatly reduced by improved terminal cleaning as they are by improved antimicrobial stewardship. This insensitivity to more stringent cleaning of environmental surfaces is shown in Table 6 by the fact that the best-ranked strategies for reducing colonizations are 27, 26, and 25, which have varying levels of effective terminal cleaning together with the most extreme antimicrobial stewardship strategies. We can conclude that Strategies 27, 26, and 25 are the best strategies for reducing both the nosocomial colonizations and infections simultaneously while Strategies 1 and 2 (baseline) are the worst for both. We also still observe, as we did in Table 5, that targeted antibiotic reduction scenario 3 (Table 3), which targets the reduction of high-risk and very high-risk antibiotics, is best at reducing nosocomial infections. Therefore, Scenarios 7, 8, 9, 16, 17, 18, 25, 26, and 27 affect the nosocomial infections the most.

**Table 6:** Parameter combinations (numbered 1-27) simulating varying levels of antimicrobial stewardship (Cols 2 and 3) and effective ward-room terminal cleaning (Col 4). The risk-level scenarios in Column 3 (numbered 1-3) are given for reference alongside the corresponding resulting change from median baseline nosocomial colonizations and infections (given in Table 4). Specific distributions of antibiotic-risk level probabilities for each risk scenario are given in Table 3

Scenario number	Half-daily antibiotic probability	Targeted antib reduction scenario	Probability of sufficient cleaning	Change from baseline median colonizations	Change from baseline median infections
1	0.27*	1*	0.2	+3.30%	+1.45%
2	0.27*	1*	0.5*	0.00%	0.00%
3	0.27*	1*	0.8	-2.26%	-2.10%
4	0.27*	2	0.2	-4.20%	-14.96%
5	0.27*	2	0.5*	-7.93%	-14.90%
6	0.27*	2	0.8	-10.09%	-17.19%
7	0.27*	3	0.2	-14.22%	-21.87%
8	0.27*	3	0.5*	-17.45%	- 23.21%
9	0.27*	3	0.8	-19.70%	-24.32%
10	0.243	1*	0.2	-6.07%	-3.27%
11	0.243	1*	0.5*	-10.04%	-5.21%
12	0.243	1*	0.8	-11.70%	-6.36%
13	0.243	2	0.2	-13.18%	-16.88%
14	0.243	2	0.5*	-16.95%	-18.37%
15	0.243	2	0.8	-18.75%	-18.25%
16	0.243	3	0.2	-22.50%	-24.83%
17	0.243	3	0.5*	-25.40%	-22.91%
18	0.243	3	0.8	-27.79%	-25.37%
19	0.216	1*	0.2	-16.70%	-5.62%
20	0.216	1*	0.5*	-19.33%	-8.45%
21	0.216	1*	0.8	-21.49%	-7.94%
22	0.216	2	0.2	- 22.44%	-18.60%
23	0.216	2	0.5*	-26.21%	- 21.21%
24	0.216	2	0.8	-27.50%	-20.97%
25	0.216	3	0.2	-31.18%	-25.25%
26	0.216	3	0.5*	-33.72%	-26.19%
27	0.216	3	0.8	-35.83%	- 25.90%

*Baseline value

Next, we consider the addition of improved HCW basic compliance to the current combinations of antimicrobial stewardship with improved terminal cleaning. To keep the number of parameter combinations under control, we select 5 of the 27 strategies listed in Table 6 to be representative of their varying effects on nosocomial colonizations and infections. To these 5 strategies (Strategies 6, 15, 18, 22, and 25), we incorporate improved HCW basic compliance. We consider three values for HCW basic compliance: 0.45 (baseline), 0.75, and 1, and we consider two values for HCW compliance with quarantined patients: 0.6 (baseline) and 1. We will consider the resulting 15 parameters combinations first with baseline HCW compliance with quarantined patients (Table 7) and then all 15 combinations again with the increased HCW compliance with quarantined patients (Table 8).

**Table 7:** Increasing levels of HCW basic compliance with non-quarantined patients (Column 5) combined with select parameter combinations from Table 6 (Scenarios 6, 15, 18, 22, and 25). The five selected scenarios varied levels of antimicrobial stewardship and ward-room terminal cleaning using a baseline level of HCW compliance with quarantined patients (60%). (Note that the decimal place in the scenario number indicates the varying levels of HCW basic compliance with non-quarantined patients while the whole number represents the original scenario number from Table 6)

Scenario number	Half-daily antibiotic probability	Targeted antib reduction scenario	Probability of sufficient cleaning	HCW basic compliance	Change from baseline median colonizations	Change from baseline median infections
6.0	0.27*	2	0.8	0.45*	-10.09%	-17.19%
6.1	0.27*	2	0.8	0.75	-19.25%	-18.59%
6.2	0.27*	2	0.8	1	-23.79%	-19.81%
15.0	0.243	2	0.8	0.45*	-18.75%	-18.25%
15.1	0.243	2	0.8	0.75	-26.92%	-20.84%
15.2	0.243	2	0.8	1	-31.10%	-21.11%
18.0	0.243	3	0.8	0.45*	-27.79%	-25.37%
18.1	0.243	3	0.8	0.75	-35.16%	-27.12%
18.2	0.243	3	0.8	1	-38.88%	-26.37%
22.0	0.216	2	0.2	0.45*	-22.44%	-18.60%
22.1	0.216	2	0.2	0.75	-29.71%	-20.95%
22.2	0.216	2	0.2	1	-34.83%	-23.00%
25.0	0.216	3	0.2	0.45*	-31.18%	-25.25%
25.1	0.216	3	0.2	0.75	-37.21%	-26.47%
25.2	0.216	3	0.2	1	-41.87%	-29.20%

*Baseline value

**Table 8:** Increasing levels of HCW basic compliance with non-quarantined patients (Column 5) combined with select parameter combinations from Table 6 (Scenarios 6, 15, 18, 22, and 25). The five selected scenarios varied levels of antimicrobial stewardship and ward-room terminal cleaning using an increased level of HCW compliance with quarantined patients (100%). (Note that the decimal place in the scenario number indicates the varying levels of HCW basic compliance with non-quarantined patients while the whole number represents the original scenario number from Table 6)

Scenario number	Half-daily antibiotic probability	Targeted antib reduction scenario	Probability of sufficient cleaning	HCW basic compliance	Change from baseline median colonizations	Change from baseline median infections
6.0	0.27*	2	0.8	0.45*	-59.83%	-30.08%
6.1	0.27*	2	0.8	0.75	-60.87%	-29.94%
6.2	0.27*	2	0.8	1	-61.21%	-27.43%
15.0	0.243	2	0.8	0.45*	-63.42%	-30.17%
15.1	0.243	2	0.8	0.75	-63.97%	-29.32%
15.2	0.243	2	0.8	1	-64.38%	-28.96%
18.0	0.243	3	0.8	0.45*	-67.39%	-33.33%
18.1	0.243	3	0.8	0.75	-67.40%	-33.56%
18.2	0.243	3	0.8	1	-67.89%	-34.81%
22.0	0.216	2	0.2	0.45*	-66.35%	-30.60%
22.1	0.216	2	0.2	0.75	-67.04%	-30.38%
22.2	0.216	2	0.2	1	-67.01%	-29.99%
25.0	0.216	3	0.2	0.45*	-69.77%	-34.04%
25.1	0.216	3	0.2	0.75	-70.16%	-33.58%
25.2	0.216	3	0.2	1	-70.25%	-34.38%

*Baseline value

The results in Table 7 show that Scenarios 25.2, 25.1, 18.2, and 18.1 are most effective at reducing nosocomial colonizations. Once again, these four scenarios implemented targeted antibiotic reduction scenario 3 (Table 3), which accounts for the most extreme reductions in high-risk and very high-risk antibiotics. The next two best scenarios, 22.2 and 15.2, surpassed Scenarios 25.0 and 18.0 (that implement targeted antibiotic reduction scenario 3 but only consider baseline HCW basic compliance with non-quarantined patients) in their effectiveness at reducing nosocomial colonizations. This is one example of a common trend we observed that often a more extreme version of one control (such as HCW basic compliance) can compensate for a less extreme version of another (such as a smaller reduction in the overall antibiotic probability). We do not, however, see this same trend when HCW compliance with quarantined patients is increased to 100% (Table 8). In this case, Scenario 25 is always better than the remaining 4 strategies for all values of HCW basic compliance with non-quarantined patients. This is expected since increasing HCW contact compliance with quarantined patients leads to less overall pathogen transfer between HCWs and rooms, so nosocomial colonizations are no longer as sensitive to changes in HCW basic compliance with non-quarantined patients.

The resulting ranking of control scenarios for reducing nosocomial infections matches what we discovered when running the control scenarios individually. All of the strategies from Tables 7 and 8 with targeted antibiotic reduction scenario 3 (18.0, 18.1, 18.2, 25.0, 25.1, 25.2) were the most effective at reducing nosocomial infections, regardless of the values of other parameters. Therefore, we still observed that a specific reduction in high-risk and very high-risk antibiotics was most effective at reducing nosocomial infections.

Next, for illustration and to reduce the number of simulations, we selected 8 scenarios from Table 7 (6.1, 6.2, 18.1, 18.2, 22.1, 22.2, 25.1, and 25.2) to which we added vaccination. The resulting combinations are numbered and labeled in Table 9. We observed that vaccination in combination with other control techniques did not change the effectiveness of those scenarios at reducing colonizations in the absence of vaccination. That is, the same scenarios we found to be most effective at reducing nosocomial colonizations in the absence of vaccination were still the most effective once vaccination was added. Furthermore, vaccination in combination with other control techniques had a similar impact on reducing nosocomial infections as it did when implemented in the absence of additional controls. Other control interventions besides vaccination would still be necessary in order to decrease *C. difficile* colonizations.

**Table 9:** Decreasing levels of immunocompromised probability (Column 6) to simulate vaccination combined with select parameter combinations from Table 7 (Scenarios 6.1, 6.2, 18.1, 18.2, 22.1, 22.2, 25.1, and 25.2). The eight selected scenarios varied levels of antimicrobial stewardship, ward-room terminal cleaning, and HCW basic compliance with non-quarantined patients while HCW compliance with quarantined patients remained at baseline (60%). (Note that the second decimal place in the scenario number indicates the varying levels of immunocompromised probabilities while XX.X represents the original scenario number from Table 7)

Scenario number	Half-daily antibiotic prob	Targeted antib reduction scenario	Prob of sufficient cleaning	HCW basic compliance	Immuno-compromised prob	Change from median baseline colonizations	Change from baseline median infections
6.1.0	0.27*	2	0.8	0.75	0.10*	-19.25%	-17.19%
6.1.1	0.27*	2	0.8	0.75	0.05	-20.19	-56.43%
6.1.2	0.27*	2	0.8	0.75	0.01	-20.88%	-89.17%
6.2.0	0.27*	2	0.8	1	0.10*	-23.79%	-19.81%
6.2.1	0.27*	2	0.8	1	0.05	-23.55%	-60.44%
6.2.2	0.27*	2	0.8	1	0.01	-23.96%	-91.83%
18.1.0	0.243	3	0.8	0.75	0.10*	-35.16%	-27.12%
18.1.1	0.243	3	0.8	0.75	0.05	-35.55%	-62.96%
18.1.2	0.243	3	0.8	0.75	0.01	-36.27%	-92.90%
18.2.0	0.243	3	0.8	1	0.10*	-38.88%	-26.37%
18.2.1	0.243	3	0.8	1	0.05	-38.85%	-64.25%
18.2.2	0.243	3	0.8	1	0.01	-39.38%	-92.83%
22.1.0	0.216	2	0.2	0.75	0.10*	-29.71%	-20.95%
22.1.1	0.216	2	0.2	0.75	0.05	-30.28%	-60.47%
22.1.2	0.216	2	0.2	0.75	0.01	-31.49%	-92.20%
22.2.0	0.216	2	0.2	1	0.10*	-34.83%	-23.00%
22.2.1	0.216	2	0.2	1	0.05	-35.05%	-61.37%
22.2.2	0.216	2	0.2	1	0.01	-35.52%	-92.23%
25.1.0	0.216	3	0.2	0.75	0.10*	-37.21%	-26.47%
25.1.1	0.216	3	0.2	0.75	0.05	-38.73%	-64.58%
25.1.2	0.216	3	0.2	0.75	0.01	41.87%	-92.38%
25.2.0	0.216	3	0.2	1	0.10*	-41.87%	-29.20%
25.2.1	0.216	3	0.2	1	0.05	-42.34%	-63.98%
25.2.2	0.216	3	0.2	1	0.01	-42.91%	-93.08%

*Baseline value

## Discussion

Systematic reviews and meta-analysis of epidemiological studies consistently conclude that antibiotic stewardship is an effective strategy against *C. difficile*, with overall reductions of *C. difficile* infections ranging from 32 to 52% depending on settings and co-implementation of control strategies [45–47]. Within stewardship practices, restrictive interventions that aim to reduce cephalosporins and fluoroquinolones were ranked as the most effective stewardship practices [45]. Similarly, our model ranked restriction of high-risk antibiotics as more effective compared with reduction on the overall antibiotic use.

Overall, antibiotic stewardship has been rarely evaluated in mathematical models for *C. difficile*; when evaluated, antibiotic stewardship was found to have low effectiveness in decreasing CDI [48]. Yacob et al. [49] reported that antibiotic stewardship was ineffective in reducing clinical diseases. Their model was an ordinary differential equations model with an overall rate of antibiotic prescription. Sensitivity analysis of similar models showed that these equation-based models were not very sensitive to the rate of antibiotic prescription [36, 50]. The discrepancy in antibiotic stewardship predictions is likely due to the difference on the representation of the level of antibiotic heterogeneity. Our results suggest that including a stratified risk of CDI is necessary to fully capture antibiotic stewardship effects.

Vaccination had a large impact on disease incidence with little impact on nosocomial colonizations since the modeled *C. difficile* toxoid vaccine does not prevent colonization, but only subsequent infection. This is also not considering a community-administered vaccine, so we are not lowering the spread in the community. We then still have the same percentage of colonized (15%) and diseased (1%) patients being admitted to the hospital and contributing to the contamination. This assessment of vaccination is preliminary as it comes with strong assumptions and is considered a best case scenario. Our tested reductions on the probability of lacking protective immunity would be equivalent to a vaccine efficacy of 50% and 90% with a 100% coverage. Although some vaccines have reached phase III clinical trials, vaccination against *C. difficile* has yet to be implemented in healthcare settings. Therefore, there are limited studies against which we can compare our results. A previous modeling assessment of vaccination predicted a 43% decrease in infections when several high-risk groups were targeted including patients with previous episodes of CDI, long-term care facility residents, and patients with planned elective surgery [51]. Achieving high efficacy may be challenging in patients lacking protective immunity and in elderly patients; therefore, future vaccine evaluation may need to consider also more modest vaccine efficacy at the individual patient level.

Within transmission-blocking interventions, increased HCW contact precaution compliance was more effective than environmental terminal cleaning. Previous ABMs for *C. difficile* had ranked transmission-blocking interventions differently. Rubin et al. [28] ranked also hand hygiene as more effective than terminal cleaning, whereas Barker et al. [30] ranked environmental cleaning as the most effective intervention. Surface-mediated transmission and transmission through contaminated hands of HCWs are tightly connected as HCWs can be contaminated by touching contaminated equipment and surfaces near patients, and surfaces can be contaminated by HCWs. ABMs are discrete-time models. Therefore, it is plausible that the order of which the events related to transmission take place may influence the relative contribution of environment versus HCW on transmission, and hence the predicted effectiveness of different transmission-blocking interventions. Barker et al. [27] summarized studies reporting bundle interventions against *C. difficile*. The studies had considerable heterogeneity in the selection of bundle components, but all studies reported a decline in CDI rates independently of the composition of the bundle [27].

The model as described has some limitations and assumptions worth noting. First, we do not allow for patients to leave their rooms. This was implemented for computational feasibility and under the assumption that the model captures additional patient exposure pathways through HCWs and their movement. The model could also offer more heterogeneity in cleaning of rooms relative to the type of patient in the room, which has potential to impact the assessment of environmental decontamination as an intervention. We could assess daily cleaning procedures in addition to the terminal cleaning currently implemented. Additionally, the model assumes that if HCWs comply with proper compliance protocol with quarantined patients, then there is a 0% chance of pathogen transfer and if not there is a 100% of transfer. The structure of this setup could explain the effectiveness of improved HCW compliance with quarantined patients and is something that could be further explored. Finally, the model was not designed to focus on details such as what types of precautions and compliance orders HCWs are implementing. Instead, our model focuses on quantifying the results of what could happen *if* HCWs are able to achieve improved overall compliance.

## Conclusion

We developed an ABM (a modification and extension of the ABM in [19]) that explicitly incorporates HCWs as vectors of transmission, tracks individual patient antibiotic histories, incorporates varying risk levels of antibiotics with respect to CDI, and tracks contamination of ward rooms by *C. difficile*. We use this ABM to simulate and evaluate the impact of different control strategies on the resulting numbers of nosocomial colonizations and infections by *C. difficile*. The control strategies considered included two forms of antimicrobial stewardship (overall reduction in antibiotics and a reduction of specifically high-risk and very high-risk antibiotics), increased environmental decontamination through terminal room cleaning, improved HCW compliance with quarantined and non-quarantined patients, and a preliminary assessment of vaccination.

Interventions that modified patient susceptibility–reduction of the use of very high- and high-risk antibiotics and vaccination–had greater effect on reducing new infections than transmission-blocking interventions. Additionally, we determined that when the control strategies are combined in various ways, a more extreme version of one control could often compensate for a less extreme version of another to effectively reduce nosocomial colonizations and infections. The resulting impact of the control scenarios on nosocomial colonizations and infections were not completely additive. In particular, the reduction in nosocomial colonizations (or infections) made by a particular combination strategy was not equal to the sum of the reduction made by the strategies individually.

## Supplementary information

**Additional file 1** Specific details about our agent-based model structure and implementation are described using the standard Overview, Design Concepts, and Details (ODD) protocol [29] and are provided as a supplemental file.

## Data Availability

Any raw data used or obtained through simulation can be shared upon request to bstephenson@lewisu.edu.

## Abbreviations

CDI: *Clostridioides difficile* Infection
HCWs: Healthcare Workers
ABM: Agent-based Model
ODD: Overview, Design Concepts, Details

## References

[1] Magill SS, O’Leary E, Janelle SJ, Thompson DL, Dumyati G, Nadle J, Wilson LE, Kainer MA, Lynfield R, Greissman S, Ray SM, Beldavs Z, Gross C, Bamberg W, Sievers M, Concannon C, Buhr N, Warnke L, Maloney M, Ocampo V, Brooks J, Oyewumi T, Sharmin S, Richards K, Rainbow J, Samper M, Hancock EB, Leaptrot D, Scalise E, Badrun F, Phelps R, Edwards JR (2018). Changes in Prevalence of Health Care-Associated Infections in U.S. Hospitals. N Engl J Med.

[2] Leffler D, Lamont T (2015). *Clostridium difficile* infection. N Engl J Med.

[3] CDC (2019). Antibiotic Resistance Threats in the United States, 2019.

[4] Lessa F, Mu Y, Bamberg W, Beldavs Z, Dumyati G, Dunn J, Farley M, Holzbauer S, Meek J, Phipps E, Wilson L, Winston L, Cohen J, Limbago B, Fridkin S, Gerding D, McDonald L (2015). Burden of *Clostridium difficile* infection in the united states. N Engl J Med.

[5] Dubberke E, Olsen M (2012). Burden of *Clostridium difficile* on the healthcare system. Clin Infect Dis.

[6] Walker AS, Eyre DW, Wyllie DH, Dingle KE, Harding RM, O’Connor L, Griffiths D, Vaughan A, Finney J, Wilcox MH, Crook DW, Peto TEA (2012). Characterisation of clostridium difficile hospital ward-based transmission using extensive epidemiological data and molecular typing. PLoS Med.

[7] Eyre DW, Cule ML, Wilson DJ, Griffiths D, Vaughan A, O’Connor L, Ip CLC, Golubchik T, Batty EM, Finney JM, Wyllie DH, Didelot X, Piazza P, Bowden R, Dingle KE, Harding RM, Crook DW, Wilcox MH, Peto TEa, Walker aS (2013). Diverse sources of C. difficile infection identified on whole-genome sequencing,. N Engl J Med.

[8] Sethi AK, Al-Nassir WN, Nerandzic MM, Bobulsky GS, Donskey CJ (2010). Persistence of skin contamination and environmental shedding of clostridium difficile during and after treatment of c. difficile infection. Infect Control Hosp Epidemiol.

[9] Galvin S, Dolan A, Cahill O, Daniels S, Humphreys H (2012). Microbial monitoring of the hospital environment: why and how?. J Hosp Infect.

[10] Morris A, Jobe B, Stoney M, Sheppard B, Deveney C, Deveney K (2002). *Clostridium difficile* colitis: an increasingly aggressive iatrogenic disease?. Arch Surg.

[11] Barlett J (2006). Narrative review: the new epidemic of *Clostridium difficile*-associated enteric disease. Ann Intern Med.

[12] Slimings C, Riley T (2014). Antibiotics and hospital-acquired *Clostridium difficile* infection: update of systematic review and meta-analysis. J Antimicrob Chemother.

[13] Dancer S, Kirkpatrick P, Corcoran D, Christison F, Farmer D, Robertson C (2013). Approaching zero: temporal effects of a restrictive antibiotic policy on hospital-acquired *Clostridium difficile*, extended spectrum *β*-lactamase-producing coliforms and methicillin-resistant *Staphylococcus aureus*. Int J Antimicrob Agents.

[14] Owens R, Donskey CJ Gaynes R, Loo C, Muto C (2008). Antimicrobial-associated risk factors for *Clostridium difficile* infection. Clin Infect Dis.

[15] McDonald LC, Gerding DN, Johnson S, Bakken JS, Carroll KC, Coffin SE, Dubberke ER, Garey KW, Gould CV, Kelly C, Loo V, Shaklee Sammons J, Sandora TJ, Wilcox MH (2018). Clinical Practice Guidelines for Clostridium difficile Infection in Adults and Children: 2017 Update by the Infectious Diseases Society of America (IDSA) and Society for Healthcare Epidemiology of America (SHEA). Clin Infect Dis.

[16] Dubberke E, Carling P, Carrico R, Donskey C, Loo V, McDonald L, Maragakis L, Sandora T, Weber D, Yokoe D (2014). Strategies to prevent *Clostridium difficile* infections in acute care hospitals: 2014 update. Infect Control Hosp Epidemiol.

[17] Feazel L, Malhotra A, Perencevich E, Kaboli P, Diekema D, Schweizer M (2014). Effect of antibiotic stewardship programmes on *Clostridium difficile* incidence: a systematic review and meta-analysis. J Antimicrob Chemother.

[18] Talpaert M, Gopal Rao G, Cooper B, Wade P (2011). Impact of guidelines and enhanced antibiotic stewardship on reducing broad-spectrum antibiotic usage and its effect on incidence of *Clostridium difficile* infection. J Antimicrob Chemother.

[19] Bintz J, Lenhart S, Lanzas C (2017). Antimicrobial stewardship and environmental decontamination for the control of *Clostridium difficile* transmission in healthcare settings. Bull Math Biol.

[20] Gerding D, Muto C, Owens R (2008). Measures to control and prevent *Clostridium difficile* infection. Clin Infect Dis.

[21] McMaster-Baxter N, Musher D (2007). *Clostridium difficile*: Recent epidemiologic findings and advances in therapy. Pharmacother J Human Pharmacol Drug Ther.

[22] Donskey C (2010). Preventing transmission of *Clostridium difficile*: Is the answer blowing in the wind?. Clin Infect Dis.

[23] Novoa A, Pi-Sunyer T, Sala M, Molins E, Castells X (2007). evaluation of hand hygience adherence in a tertiary hospital. Am J Infect Control.

[24] Codella J, Safdar N, Heffernan R, Alagoz O (2014). An agent-based simulation model for *Clostridium difficile* infection control. Med Dec Making.

[25] de Bruyn G, Saleh J, Workman D, Pollak R, Elinoff V, Fraser NJ, Lefebvre G, Martens M, Mills RE, Nathan R, Trevino M, van Cleeff M, Foglia G, Ozol-Godfrey A, Patel DM, Pietrobon PJ, Gesser R, Arslanian A, Gagianas P, Brookmyre A, Christensen S, Cook R, Earl J, Eder F, Evans P, Glover R, Haselby R, Henry D, Hoekstra J, Radin D, Rosen J, Rubino J, Segall N, White A, Yangco B, Zakko S, Ervin J, Casanova R, Griffin C, Weiner G, Barish C, Kapoor O, Fernandez M, Epstein R, Poling T, Lesh K, Challa V, Farrington C, Bauch T, Chen M, Arora S (2016). Defining the optimal formulation and schedule of a candidate toxoid vaccine against Clostridium difficile infection: A randomized Phase 2 clinical trial. Vaccine.

[26] Hermes-DeSantis E, Henderson M, Shah M, Bragg A, Fahim G (2017). A Review of the Safety and Efficacy of Vaccines as Prophylaxis for Clostridium difficile Infections. Vaccines.

[27] Barker AK, Ngam C, Musuuza JS, Vaughn VM, Safdar N (2017). Reducing clostridium difficile in the inpatient setting: a systematic review of the adherence to and effectiveness of c. difficile prevention bundles. Infect Control Hosp Epidemiol.

[28] Rubin M, Jones M, Leecaster M, Khader K, Ray W, Huttner A, Huttner B, Toth D, Sablay T, Borotkanics R, Gerding D, Samore M (2013). A simulation-based assessment of strategies to control *Clostridium difficile* transmission and infection. PLoS ONE.

[29] Railsback S, Grimm V (2012). Agent-based and Individual-based Modeling: a Practical Introduction.

[30] Barker AK, Alagoz O, Safdar N (2018). Interventions to reduce the incidence of hospital-onset clostridium difficile infection: an agent-based modeling approach to evaluate clinical effectiveness in adult acute care hospitals. Clin Infect Dis.

[31] Lanzas C, Dubberke ER (2014). Effectiveness of screening hospital admissions to detect asymptomatic carriers of clostridium difficile: a modeling evaluation. Infect Control Hosp Epidemiol.

[32] Grimm V, Berger U, DeAngelis D, Polhill J, Giske J, Railsback S (2010). The ODD protocol: a review and first update. Ecol Model.

[33] Wilensky U (1999). Center for connected learning and computer-based modeling.

[34] Jinadatha C, Villamaria F, Coppin J, Dale C, Williams M, Whitworth R, Stibich M (2017). Interaction of healthcare worker hands and portable medical equipment: a sequence analysis to show potential transmission opportunities. BMC Infect Dis.

[35] Suwantarat N, Supple L, Cadnum J, Sankar T, Donskey C (2017). Quantitative assessment of interactions between hospitalized patients and portable medical equipment and other fomites. Am J Infect Control.

[36] Lanzas C, Dubberke E, Lu Z, Reske K, Grohn Y (2011). Epidemiological model for *Clostridium difficile* transmission in healthcare settings. Infect Control Hosp Epidemiol.

[37] Gilboa M, Hour-Levi E, Cohen C, Tal I, Rubin C, Feld-Simon O, Brom A, Eden-Friedman Y, Segal S, Rahav G, Regev-Yochay G. Environmental shedding of toxigenic *Clostridioides difficile* by asymptomatic carriers: A prospective observational study. Clin Microbiol Infect. 2020. 10.1016/j.cmi.2019.12.011. 10.1016/j.cmi.2019.12.011 31904567

[38] Stephenson B, Lanzas C, Lenhart S, Day J (2017). Optimal control of vaccination in an epidemiological model of *Clostridium difficile* transmission. J Math Biol.

[39] Alasmari F, Seiler S, Hink T, Burnham C, Dubberke E (2014). Prevalence and risk factors for asymptomatic *Clostridium difficile* carriage. Clin Infect Dis.

[40] Kachrimanidou M, Malisiovas N (2011). *Clostridium difficile* infection: a comprehensive review. Crit Rev Microbiol.

[41] Kim PW, Roghmann M-C, Perencevich EN, Harris AD (2003). Rates of hand disinfection associated with glove use, patient isolation, and changes between exposure to various body sites. Am J Infect Control.

[42] Erasmus V, Daha TJ, Brug H, Richardus JH, Behrendt MD, Vos MC, van Beeck EF (2010). Systematic review of studies on compliance with hand hygiene guidelines in hospital care. Infect Control Hosp Epidemiol.

[43] Ponce E, Stephenson B, Lenhart S, Day J, Peterson GD. Papas: A portable, lightweight, and generic framework for parallel parameter studies. 2018:47–1478. 10.1145/3219104.3229289.

[44] Crobach M, Vernon J, Loo V, Kong L, Pèchinè S, Wilcox M, Kuijper E (2018). Understanding *Clostridium difficile* colonization. Clin Microbiol Rev.

[45] Feazel LM, Malhotra A, Perencevich EN, Kaboli P, Diekema DJ, Schweizer ML (2014). Effect of antibiotic stewardship programmes on clostridium difficile incidence: a systematic review and meta-analysis. J Antimicrob Chemother.

[46] Baur D, Gladstone BP, Burkert F, Carrara E, Foschi F, Döbele S, Tacconelli E (2017). Effect of antibiotic stewardship on the incidence of infection and colonisation with antibiotic-resistant bacteria and clostridium difficile infection: a systematic review and meta-analysis. Lancet Infect Dis.

[47] Hsu J, Abad C, Dinh M, Safdar N (2010). Prevention of endemic healthcare-associated clostridium difficile infection: reviewing the evidence. Am J Gastroenterol.

[48] Gingras G, Guertin M-H, Laprise J-F, Drolet M, Brisson M (2016). Mathematical modeling of the transmission dynamics of clostridium difficile infection and colonization in healthcare settings: a systematic review. PloS one.

[49] Yakob L, Riley TV, Paterson DL, Marquess J, Clements AC (2014). Assessing control bundles for clostridium difficile: a review and mathematical model. Emerg Microbes Infect.

[50] Yakob L, Riley TV, Paterson DL, Clements AC (2013). Clostridium difficile exposure as an insidious source of infection in healthcare settings: an epidemiological model. BMC Infect Dis.

[51] van Kleef E, Deeny SR, Jit M, Cookson B, Goldenberg SD, Edmunds WJ, Robotham JV (2016). The projected effectiveness of clostridium difficile vaccination as part of an integrated infection control strategy. Vaccine.

