# Supplementary material for: Comparing intervention strategies for reducing Clostridioides difficile transmission in acute healthcare settings: an agent-based modeling study
Source: BMC Infect Dis. 2020 Oct 28;20:799. doi: 10.1186/s12879-020-05501-w (PMC7594474; doi:10.1186/s12879-020-05501-w)
Supplement: Supplementary file 1 — Additional file 1 Specific details about our agent-based model structure and implementation are described using the standard Overview, Design Concepts, and Details (ODD) protocol [29] and are provided as a supplemental file. [file 12879_2020_5501_MOESM1_ESM.pdf]

Additional File 1

Comparing Intervention Strategies for Reducing Clostridioides difficile Transmission in Acute Healthcare Settings: An Agent-Based Modeling Study

Brittany Stephenson[1], Cristina Lanzas[2], Suzanne Lenhart[3], Eduardo Ponce[4], Jason Bintz[5], Erik R. Dubberke[6] and Judy Day[3,4]

1 ODD Protocol: Overview

In this section, we describe the Overview, Design concepts, and Details (ODD) of our agent-based model (ABM) to provide a more complete and rigorous model description. ODD was designed by experienced modelers to create a complete, quick, and exhaustive method for describing ABMs for the purpose of standardization and replication [1].

1.1 Purpose

We modified and expanded the agent-based model in [2], which simulates the transmission of *Clostridioides difficile* in a healthcare setting. In particular, we explicitly incorporated healthcare workers as vectors of transmission and then evaluated the following: (1) the efficacy of control measures such as antimicrobial and environmental stewardship and (2) the impact of healthcare workers (HCWs) on the spread of nosocomial *C. difficile* infection (CDI).

1.2 Input data

The agent behaviors, parameter values, and initial conditions are based on information from either published literature or data collected from six medicines wards at Barnes-Jewish Hospital in St. Louis, Missouri. Information about the collection of this data is described by Lanzas et al. in [3]. We also incorporated updated hospital data that was collected after the publication of [3], such as an increased admission of colonized patients, described by Alasmari et al. [4].

By varying model inputs, we simulated and compared the impact of a variety of control measures on reducing nosocomial colonization and infection. The first such strategy involved varying the probability of effective cleaning. As in the original ABM in [2], we defined effective cleaning to be “cleaning that reduces the contamination level of a ward room." The cleanliness, or lack thereof, of each ward room affects the probability that a patient will become colonized, so increasing the probability of effective cleaning serves as a disease control measure.

Because HCWs are the only agents that move from room to room, they are important vectors of spore transmission, so our second control strategy involved varying HCW basic compliance with non-quarantined patients and HCW contact compliance specifically with CDI patients. *HCW-basic-compliance* is defined as the level of compliance that HCWs adhere to when following protocol after visiting patients.

Our baseline value for *HCW-basic-compliance* was based on the values given by Rubin et al. in [5]. We define *HCW-compliance-CDI-patients* to be the level of compliance that HCWs adhere to when visiting a quarantined patient with CDI. Data indicates that HCWs are more compliant with hand-washing and other contact precautions when they know they are visiting an infected patient. We set the baseline value of this variable to be 0.6 to match the data for percentage of adherence to the use of contact precautions in isolation rooms given in [5].

Antimicrobial stewardship was determined in [2] to be another important control measure. One method of implementing antimicrobial stewardship involves reducing the overall number of antibiotics given to patients. We implement this in the same way that Bintz et al. do in [2]: define q to be the proportion by which we want to reduce antibiotic treatments in the hospital. Then, out of all patients who will receive an antibiotic at each time step, determined by the probability of receiving an antibiotic, only $1 - q$ of them will now receive an antibiotic. A second method of antimicrobial stewardship involves altering the relative proportions of antibiotics given. As in [2], we define three risk levels of antibiotics prescribed (low, high, or very high) based on whether they make patients more or less at risk of contracting CDI. The baseline values for the probability of receiving a low-risk, high-risk, or very-high-risk antibiotic (0.4, 0.26, and 0.34, respectively) were taken from the data and are the same as those used in [2]. Prevention strategies considered involve decreasing the proportion of very-high-risk antibiotics prescribed.

1.3 Entities, state variables, and scales

Our ABM has three entities: patients, healthcare workers, and ward rooms. The model was designed to mimic characteristics of Barnes-Jewish Hospital, from where the original data was obtained. As such, we considered a hospital with six medical wards of 35 rooms each, with at most one patient occupying a room at any given time. This allowed for a total possible capacity of 210 patients, and we set the baseline occupancy level to be 0.85. Patient state variables are updated on a half-day time scale, which reflects the time step used in [2]. HCW state variables are updated every 15 minutes to reflect their movement from room to room.

Room State Variables Each ward room is characterized by four state variables, listed in Table 1. First, each room is assigned to a specific ward. Next, all rooms have a contamination level that is updated on the 15-minute time step scale. HCWs can increase the contamination level of a room upon entering, depending on *prob-hcw-transfer*, the probability that an HCW will transfer pathogen to the room, described more in Section 2.9. Each half-day symptomatic and asymptomatic patients contribute to the contamination level of a room. Effective cleaning can reduce the contamination level of a room, and cleaning is implemented each half day only after a patient is discharged from that room. The contamination level of an individual room affects the probability that a susceptible patient in that room will become colonized.

The next state variable that governs room characteristics is whether or not a room is occupied by a patient under quarantine. According to [6], symptomatic patients are placed in quarantine to ensure proper contact precautions are implemented. Finally, a room is characterized by the state variable *prob-room-transfer*.

Room variable	Description	Possible value(s)
ward-number	Specifies in which ward the room is located	1, 2, ..., 6
contamination-level	Tracks the amount of contamination in room	$[0, \infty)$
quarantine-patient-here	Specifies whether or not current occupant is under quarantine	yes, no
prob-room-transfer	Gives the probability the room will transfer pathogen to an HCW	$[0, 1]$

Table 1: Room state variable explanations and values

This probability is updated each 15-minute time step and determines the likelihood of an HCW picking up pathogen upon entering that particular room. More information about determining this probability is also described in Section 2.9.

Patient State Variables Patient behavior is characterized by many state variables, all of which are listed in Table 2. A patient's disease status is tracked beginning at admission. A patient can either be resistant to contracting CDI, susceptible to contracting CDI, colonized by *C. difficile* (asymptomatic), or diseased (symptomatic CDI). Because taking an antibiotic is the strongest risk factor for contracting CDI [7], our assumption is that any patient who has not received an antibiotic is resistant to CDI. In particular, it has been shown that more than 90% of patients of hospitalized patients with CDI recently underwent antimicrobial therapy [8]. Once an antibiotic is given, a patient becomes susceptible to colonization. Depending on a patient's probability of becoming colonized, a susceptible patient who comes into contact with *C. difficile* spores may become colonized and, therefore, become an asymptomatic carrier.

A patient either will or will not be lacking protective immunity. A patient lacking protective immunity is a colonized patient who is unable to mount his or her own immune response and, therefore, will contract CDI. Those who are not lacking protective immunity are able to mount an immune response and will not experience CDI symptoms. Each susceptible and asymptomatically colonized patient has a probability of his or her gut microbiota returning to normal (and therefore regaining resistance). If a colonized patient is not lacking protective immunity and receives an antibiotic, it is possible to revert to being susceptible; if he or she is lacking protective immunity and does not receive an antibiotic, it is possible to regain resistance. A colonized patient lacking protective immunity who receives an antibiotic will contract CDI more quickly than patients lacking protective immunity who do not receive antibiotics.

Each patient that is admitted is assigned a length of stay based on his or her disease status. Patients' time since admission and time since current disease status are both tracked while, for patients who have received an antibiotic, their time since beginning antibiotics is tracked in addition to the risk level (low, high, or very high) of the antibiotic taken and the number of antibiotics taken. Diseased patients may or may not be identified as diseased upon screening, and their time since a successful screening is tracked. Additionally, diseased patients who are successfully identified may or may not be treated successfully, and their time since beginning treatment is tracked.

Patient variable	Description	Possible value(s)
disease-status	Tracks patient disease status	R, S, C, D
disease-status-at-admission	Identifies patient disease status upon admission	R, S, C, D
length-of-stay	Specifies a patient's length of stay in the hospital (half-days)	[0, 160]
time-since-admission	Tracks amount of time since patient was admitted (half-days)	0, 1, 2, ...
time-since-current-disease-status	Tracks amount of time since patient current disease status began	0, 1, 2, ...
immunocompromised	Indicates whether or not a patient will mount an immune response to colonization	yes, no
treatment-length	Gives prescribed length of current antibiotic treatment (half-days)	14
time-to-normal	Gives time until patient's gut microbiota returns to normal (half-days)<br>low-risk antibiotic<br>high-risk antibiotic<br>very-high-risk antibiotic	 <br>28<br>28<br>70
time-since-began-antib	Tracks time since patient began current antibiotic treatment (half-days)	0, 1, 2, ...
prob-regaining-resistance	Gives the probability of regaining resistance to colonization	$[0, 1]$
prob-becoming-colonized	Gives the probability of a susceptible patient becoming colonized	$[0, 1]$
antib-risk-level	Indicates the risk level of the current antibiotic with respect to CDI	low, high, very-high
number-hosp-antibs	Tracks the number of antibiotics a patient has received	0, 1, 2, ...
length-incubation-period	Gives the length of time between colonization and becoming diseased (half-days)<br>low-risk antibiotic<br>high-risk antibiotic<br>very-high-risk antibiotic	 <br>$[20, 60]$<br>$[14, 40]$<br>$[8, 20]$
time-until-diseased	Gives the length of time until a colonized patient lacking protective immunity becomes diseased (half-days)	$[0, 60]$
will-ID	Indicates whether a screening will correctly test positive for CDI	yes, no
time-since-succ-screen	Tracks the amount of time since patient correctly tested positive for CDI (half-days)	0,1,2...
will-treat-succ	Determines whether a patient will be treated for CDI successfully	yes, no

Table 2: Patient state variable explanations and values

HCW variable	Description	Possible value(s)
shift-length	Specifies length of HCW's shift (hours)	8, 12
time-since-shift-began	Tracks the time since an HCW began a shift (hours)	$[0, 12]$
carrier-level	Tracks amount of contamination on an HCW's hands	$[0, \infty)$
hcw-type	Indicates whether an HCW will be Type 1 or Type 2	Type 1, Type 2
hcw-risk	Indicates the risk level of the task an HCW is performing	low, medium, high
prob-hcw-transfer	Gives the probability an HCW will transfer pathogen to a room	$[0, 1]$

Table 3: HCW state variable explanations and values

Healthcare Worker State Variables HCWs are initially assigned a ward, and it is assumed HCWs visit only patients in that same ward for the entirety of their shift. Each HCW is assigned a shift length upon entry to the hospital, and an HCW's time since beginning a shift is tracked. There is a 50% chance an HCW will have an 8-hour shift and a 50% chance an HCW will work a 12-hour shift. Healthcare workers are divided into two types: Type 1 HCWs visit many patients for short periods of time (assumed to be 15 minutes), and Type 2 HCWs visit fewer patients for longer periods of time (assumed to be 45 minutes). Type 1 HCWs move systematically from room to room, meaning that they move to the next closest occupied room at each 15-minute time step. Type 2 HCWs move randomly from room to room within the same ward. It is assumed there will never be more than one Type 1 HCW or more than one Type 2 HCW in a given room simultaneously; however, there can be a Type 2 and a Type 1 HCW visiting the same patient at the same time. HCWs will never enter rooms not occupied by a patient.

All HCWs have individual contamination levels similar to the contamination levels tracked for each ward room. We refer to HCWs' contamination levels as their *carrier-level*, which represents the amount of pathogen on their hands. For simplicity, we assume that all HCWs begin their shifts with carrier levels of zero; however, this could soon need to be increased as data indicates community-associated CDI is increasing [9]. Once in the hospital, HCW carrier levels can be increased upon picking up pathogen in contaminated rooms and can be decreased by adherence to proper hand-washing and contact precaution protocols. Each HCW has a probability of transferring pathogen to a room upon entry, referred to as *prob-hcw-transfer* (Section 2.9).

Not only does the time HCWs spend with patients vary, but also the type of task they perform on a patient can affect their probability of picking up *C. difficile* spores. We define three task levels (low risk, medium risk, or high risk) based on the risk of transfer. HCWs can perform any risk level task, but we assume Type 1 HCWs are more likely to perform low-risk tasks while Type 2 HCWs are more likely to perform high-risk tasks. A list of all HCW variable values along with explanations is given in Table 3.

Global State Variables A summary description of all global variables is given in the original manuscript. Admission proportions for each disease status (a_r, a_s, a_c, and

a_d) are global variables based on the Barnes-Jewish Hospital data detailed in [3] with modifications made based on the updated data in [4]. This new data indicated that 15% of admitted patients were colonized upon arrival, so we updated a_c and thereby decreased a_s to reflect this. Thus, we set the probability of a patient being colonized upon admission to 0.15, the probability of a patient being susceptible upon admission to 0.09, the probability of a patient being resistant upon admission to 0.75, and the probability of a patient having CDI upon admission to 0.01.

The probability of an HCW complying with proper basic protocol after leaving a room has a baseline value of 0.45. In particular, we averaged the percentage of hand-hygiene adherence for nurses and doctors in non-isolated rooms given in [5]. The probability an HCW who has just visited a quarantined patient will follow proper protocol is set slightly higher to 0.6 to match the percentage of adherence to the use of contact precautions in isolation rooms given in [5]. The percentage of total contamination that an HCW will transfer to a room after determining transfer will occur is a global variable set to 90% while the percentage of pathogen a room will transfer to an HCW is set to 10%. Assessments of hospital cleaning practices have shown that routine cleaning results in decontamination of no more than 56% of targeted surfaces [10]. If a room is effectively cleaned, we set the contamination level to be reduced by 50%.

The probability of susceptible patients becoming colonized depends on both the contamination level of their room and the risk level of their current antibiotic. Similar to antibiotic risks, room contamination levels are divided into three groups (low, medium, or high contamination). Rooms with contamination levels between 0 and 0.4 are considered low contamination while rooms with contamination levels between 0.4 and 0.8 are considered to have medium contamination. Any rooms with contamination levels higher than 0.8 are considered highly contaminated. Each probability of becoming colonized is denoted with a subscript referring to the antibiotic-risk level and a superscript referring to the room-contamination level. For example, p_l^h is the probability a patient on a low-risk antibiotic becomes colonized in a highly contaminated room. Probabilities for each combination of antibiotic-risk level and room-contamination level are denoted in a similar way. Values for each of these probabilities and for the room-contamination cut-off levels were chosen so that nosocomial colonizations accounted for 20% of admissions [11, 12]. More details about these calculations are given in Section 2.13.

All global variables described in the remainder of this section are kept the same as those used in the original ABM in [2]. We define the hospital occupancy level to be a global variable that is set to 0.85. The probability a patient is lacks protective immunity is a global variable with baseline value 0.1. To determine the probability that a susceptible or colonized patient with protective immunity will regain resistance, we define a global variable for the minimum probability of regaining resistance, p_{rrmin}. At each half-day, there is a 27% chance a patient will begin antibiotic therapy. Bintz et al. [2] chose this value as the baseline so that the simulated total number of antibiotic treatments per patient matched the data. The baseline values for the probabilities of receiving a low-risk, high-risk, or very-high-risk antibiotic were taken from the dataset to be 0.4, 0.26, and 0.34, respectively.

The probability of effectively cleaning a room is set to a baseline value of 0.5,

described more in Section 2.6. Symptomatic patients are screened for CDI, and the sensitivity of this test is a global variable set to 0.91. The turnover time for this test is assumed to be 2 half days [13]. The probability of successfully treating a patient with CDI has a baseline value of 0.8, based on the dataset from Barnes-Jewish Hospital.

1.4 Process overview and scheduling

Our model runs some processes with a 15-minute time step and other processes with a half-day time step. The following HCW processes occur every 15 minutes: first, some HCWs may begin a new shift, and all HCWs will perform tasks on patients. Type 1 HCWs will move from room to room every 15 minutes, and Type 2 HCWs will move every 45 minutes (3 time steps). Once an HCW enters a room, the risk level of the task he or she will perform is determined. Then, the probability of an HCW transferring pathogen to a room and the probability of a room transferring pathogen to an HCW are determined. Based on these probabilities, HCW carrier levels and room contamination levels are updated. After HCWs leave a room, they can reduce their contamination levels if they have properly followed protocol. Finally, a shift change occurs, and those who have finished their shift will leave the hospital before the process starts again. After the shift change, HCW time characteristics are updated.

Patient behaviors and interactions are updated each half-day in the following order: patients are admitted, their disease status is updated, the room contamination levels are then updated based on contributions from asymptomatic and symptomatic patients, and then patients may be discharged. Upon discharge, vacant rooms are then cleaned. Lastly, time characteristics are updated for the patients.

1.5 Initialization

The hospital is initially populated by patients whose disease statuses are based on the proportions a_r, a_s, a_c, and a_d given in global variables table of the original paper. There are 210 available rooms that are filled to 85% occupancy. This occupancy proportion remains constant because we set the number of patients admitted each half-day equal to the number of patients discharged in the previous half-day. The number of HCWs is chosen so that there is a 3:1 ratio of patients to HCWs, and this is kept constant by assuming the number of HCWs who begin their shifts at each 15-minute time step equals the number of HCWs who left at the last time step. Initial room contamination levels are based on the disease status of the patient in the room upon initialization, and the initial carrier level of all HCWs is assumed to be zero. The shift-lengths of HCWs who initially populate the model are set to random lengths between 0 and 12 hours since we assume that not all of them arrived to work at the same time. We simulate a three-week time period before recording any outputs to ensure that initial conditions are not significantly affecting the results.

2 Submodels

In this section, we detail all the submodels that together form the overall simulation routine.

2.1 Admit HCWs

At the beginning of each 15-minute time step, new HCWs arrive to replace those who left the hospital at the end of the previous time step. The number who arrive is set equal to the number who left at the previous time step to ensure that the ratio of 1 HCW to every 3 patients is maintained. Upon arrival, each HCW is randomly assigned to a ward, and he or she remains in that same ward for the entirety of the shift. HCWs are only initially admitted into patient-occupied rooms where no other HCWs are currently present.

Once a new HCW arrives to the hospital, he or she is assigned a Type 1 or Type 2 role with a 50% chance of each. Additionally, a HCW's pathogen level is set to zero upon arrival, and he or she is assigned a length of shift. For simplicity, we consider two possible shift lengths: 8 hours or 12 hours. There is a 50% chance a HCW will work an 8-hour shift, and a 50% chance he or she will work a 12-hour shift; this is decided for each HCW once arriving to the hospital.

2.2 Admit patients

This subroutine is modeled in the same way as that described in [2] with small modifications. At the beginning of each half-day time step, new patients are admitted to replace those discharged at the end of the previous time step. Similar to the arrival of HCWs, the number of new patients admitted equals the number of patients discharged at the previous time step to maintain a constant total population. Because of the variability in the number of patients discharged at the end of each time step, this also ensures consistency and keeps the number of patients lower than the total number of ward rooms. The assignment of disease status upon admission is determined with the same probabilities used in the initialization process: a_r, a_s, a_c, and a_d. That is, there is a 75% chance an admitted patient is resistant [3], a 9% chance an admitted patient is susceptible, a 15% chance an admitted patient is colonized [4], and a 1% chance an admitted patient has CDI [3]. After a patient is assigned a disease status, we set his or her time since entering this disease status to zero so that we can track this throughout the patient's hospital stay. The only exception to this is for resistant patients; we do not track their time since becoming resistant since this time does not affect their probability of moving to another disease class or how long it will be until they move to another disease class.

When patients are admitted, we initialize their number of antibiotics received in the hospital to zero, and their time since admission is also set to zero. Patients are randomly assigned a room upon admission and will only be assigned to vacant rooms since we assume all hospital rooms are single patient rooms. Also upon arrival, each patient is assigned a length of stay, which is determined by the subroutine described in Section 2.16.

When a patient is admitted as susceptible, we first assign that patient an antibiotic history because of our assumption that the only way to become susceptible to colonization is through the disruption of the normal gut microbiota by antibiotics. The process for determining the type of antibiotic that we will assign is described in Section 2.12. We next assign the susceptible patient a time since beginning antibiotic treatment (which will vary since antibiotic treatment began prior to entering the hospital). As described in [2], we set this time to "a random integer drawn from

a uniform distribution ranging from 0 to an upper limit defined as the sum of the treatment length (14 half-days) and time until microbiota recovery (28 half-days for low- and high-risk antibiotics and 70 half-days for very-high-risk antibiotics)." This mimics our assumption that patients become susceptible immediately after receiving antimicrobial treatment and remain susceptible until the restoration of their normal gut microbiota. Lastly, a susceptible patient's time since becoming susceptible is set equal to his or her time since beginning an antibiotic.

Upon admission of a colonized patient, we first must determine whether or not this patient lacks protective immunity. We do so by using the global variable for the probability a colonized patient will not mount an immune response. Once it is determined that a patient is not lacking protective immunity, we then assign the patient an antibiotic, using the subroutine described in Section 2.12. Next, we assign a time since beginning antibiotics in the same way described in the previous paragraph for susceptible patients. Because these patients can become colonized anytime after receiving an antibiotic, we lastly set their time since becoming colonized to be a random integer chosen from a uniform distribution ranging from 0 to their time since beginning antibiotics.

If it is determined that a colonized patient is lacking protective immunity upon admission, this patient will contract CDI, and the time until doing so is referred to as the incubation period. The length of the incubation period for a particular patient depends on the risk level of the antibiotic assigned. Therefore, for each patient lacking protective immunity, we begin by assigning an antibiotic (Section 2.12). The minimum and maximum possible lengths of the incubation period for various antibiotic-risk levels are given in Table 2. In particular, for those patients assigned a low-risk antibiotic, their incubation period is assigned to be a random integer from a uniform distribution over 20 to 60 half-days. Patients on a high-risk antibiotic have a greater chance of contracting CDI more quickly, so they are assigned an incubation period ranging from 14 to 40 half-days while patients on very-high-risk antibiotics are assigned an incubation period between 8 and 20 half-days. Once a patient lacking protective immunity is assigned an incubation period, he or she is then assigned a time until becoming diseased, which we set to a random integer less than or equal to the incubation period. Lastly, we track their time since becoming colonized by setting it equal to the length of the incubation period minus the time until becoming diseased, as described in [2].

If a patient is diseased upon admission, we begin by setting his or her time since becoming diseased to be a random integer less than or equal to 21 half-days, as used in [2]. We then assign whether or not each diseased patient will be treated successfully by assigning the global variable for the probability of successful treatment as 0.8 [14]. Next, we decide if the hospital will successfully identify a diseased patient as diseased upon screening. Note that all patients are screened upon admission and/or upon becoming diseased [2]. The success of the screening depends on the sensitivity of the test for CDI, which we represent with the sensitivity global variable given as 0.91 [13]. If a patient will be successfully identified as diseased, we initialize his or her time since a successful screening to zero. Similarly, we will set the time since an unsuccessful screening to zero for those patients who are not successfully identified. The turnaround time for the test is set to be 2 half-days on average [13]; once a

patient's time since a successful screening reaches this turnaround time, he or she will be quarantined and begin treatment for CDI, a procedure described in Section 2.15. Those diseased patients who were unsuccessfully screened for CDI will not be tested again until after the turnaround time has passed.

2.3 Update disease status

This subroutine is run at each half-day time step and is implemented similarly to the update-disease-status procedure described in [2]. There is a global variable for the probability that a patient will receive an antibiotic each half-day. In [2], they determined this probability should be 0.27 so that the total number of antibiotic treatments per patient matched the data from Barnes-Jewish Hospital. All possible disease-status transitions for a patient were briefly described in Section 1.3. In this section, we describe in more detail the transitions and how they are implemented.

Once patients transition to a new disease status, their time since entering this new status is set to 0. A resistant patient's only possible movement is to the susceptible class by taking an antibiotic. If it is determined that a resistant patient receives an antibiotic, then that patient will be assigned an antibiotic (Section 2.12). We also keep track of the number of antibiotics a patient receives while in the hospital, so we update this number here. Next, an updated length of stay will be determined based on the patient's new status as susceptible; more details about this are given in Section 2.16. If this updated length of stay is greater than the current length of stay assigned to the patient, then the patient's current length of stay will be modified to reflect the longer length of stay.

A susceptible patient can either move back to the resistant class or become colonized after being exposed to pathogen. For each susceptible patient, this subroutine begins by determining his or her probability of regaining resistance, which varies at each half-day time step. Details about the calculation of this probability are given in Section 2.14. Based on this probability, a susceptible patient may return to the resistant class. If a susceptible patient does not return to resistant, there is a 27% chance that he or she will receive an additional antibiotic, and the type of antibiotic received is determined by the procedure described in Section 2.12.

Each susceptible patient also has a probability of becoming colonized that depends on the local contamination level and on the risk level of the antibiotic(s) received. Details about the calculation of this probability are given in Section 2.13. We also keep track of the number of patients who become colonized while in the hospital. If it is determined a susceptible patient will become colonized, we count him or her in this list and then determine whether or not he or she will be lacking protective immunity. For those who are lacking protective immunity, their incubation period is set in the same way as described in the admit-patients subroutine (Section 2.2).

For those colonized patients who are not lacking protective immunity, they also have a probability of regaining resistance (Section 2.14), or, like susceptible patients, they may receive an additional antibiotic. If so, their total number of hospital antibiotics and time since beginning an antibiotic are updated to reflect this.

Colonized patients who are lacking protective immunity also have a chance of receiving an additional antibiotic. Once the type of antibiotic is determined, this additional antibiotic may decrease their incubation period. In particular, for a high-risk antibiotic, their time until becoming diseased is decreased by 10%, and for a

very-high-risk antibiotic, their time until becoming diseased is decreased by 20%. Once their time until becoming diseased reaches 0, they move to the disease class and receive an updated length of stay based on this new disease status (Section 2.16). Now that these patients are symptomatic, they will be screened at the next half-day time step. We then determine whether these patients will be treated and/or screened successfully (in the same manner as described in Section 2.2). These patients are then counted in the number of patients who become diseased while in the hospital.

The only possible transition for diseased patients is back to the susceptible class. If a diseased patient had a successful screening, he or she will be quarantined and begin treatment once the turnover time for the screening test has elapsed. If it is determined that the treatment will be successful, then the diseased patient may move back to the susceptible class after 20 half-days [2]. If a diseased patient had an unsuccessful screening, then after the turnover time has elapsed, he or she will be re-screened for CDI.

2.4 Update room contamination levels

After patient disease statuses are updated, the model updates the room contamination levels based on the disease status of the patient in the room. This particular subroutine only considers patient contributions to overall room contamination levels; HCW contributions are updated every 15 minutes using a different submodel (Section 2.9). A colonized patient will contribute an amount in the range $[1, 2)$ to the room contamination level at each half-day time step. Because the transmission potential is higher for patients with active disease than in asymptomatic carriers [15], a diseased patient will contribute an amount in the range $[2, 3)$ at each half-day time step. Note that these ranges are larger than the possible ranges for HCW contributions since HCW contributions are updated at every 15-minute time step.

2.5 Discharge patients

Once a patient's time since being admitted reaches his or her length of stay (assigned upon admission based on the procedure described in Section 2.16), that patient is discharged from the hospital. Discharges can only occur at each half-day time step. In the discharge subroutine, we also tally the total number of patients discharged, the total number of patients discharged for each disease status, and the total number of antibiotics given in the hospital. The average length of stay for all discharged patients is also calculated.

2.6 Clean ward rooms

After patients are discharged, their vacant room is cleaned. The probability the room will be effectively cleaned is a global variable with a value of 0.5 [2]. This baseline value was chosen because, depending on which cleaning measures are used, the cleaning could be more or less effective, and this probability can be adjusted to mimic more or less intensive cleaning. Our model only explicitly accounts for terminal cleaning; however, daily cleaning of select rooms could be implemented in the case of an outbreak. This can be incorporated in our model by modifying the contamination level of rooms that were targeted by extra cleaning.

In this subroutine, if it is determined a room will be effectively cleaned, the contamination level of the room will decrease by 50%, which is represented by the global variable *clean-reduction*. Similar to the probability of effective cleaning, this percentage can be increased to model more targeted and intensive cleaning.

2.7 Update patient time characteristics

Patient time characteristics are updated at each half-day time step. This includes their time since being admitted and, for all patients except resistant patients, their time since entering their current disease status. As mentioned in Section 2.2, we do not track this time for resistant patients because it does not affect whether or not they will become susceptible; this is only determined by their probability of receiving an antibiotic.

For susceptible and colonized patients (not lacking protective immunity), this submodel increases their time since beginning an antibiotic by one half-day time step each time it is run. For the colonized patients lacking protective immunity, their time until becoming diseased is decreased by one half-day. We also update the time since a diseased patient has received a successful, or unsuccessful, screening for CDI, and for those diseased patients who have begun treatment, we update their time since beginning treatment.

2.8 HCW movement

This subroutine defines how HCWs move from room to room, which depends on whether or not they are Type 1 HCWs or Type 2 HCWs. HCWs are instructed to only move into rooms that are currently occupied by a patient and will never enter vacant ward rooms. We also assume that all HCWs remain in the same ward for the entirety of their shift and never move from ward to ward.

In each ward, the rooms are numbered from 1 to 35. Type 1 HCWs, skipping any rooms that are unoccupied, move every 15-minute time step from one room to the next in order of room number. Upon reaching room 35, a Type 1 HCW will next move to room 1 of that ward (as long as it is occupied at that time). Type 2 HCWs move randomly within a ward, and only move every three 15-minute time steps. It is not possible for two Type 1 HCWs to be in the same room at the same time or for two Type 2 HCWs to be in the same room at the same time; however, it is possible for a Type 1 and a Type 2 to visit the same patient at the same time.

2.9 Update HCW and room contamination levels

This subroutine defines both how an HCW will transfer pathogen *to* a room and how an HCW will pick up pathogen *from* a room. It has three parts: defining risk level of tasks being performed, determining the probability of transfer, and updating contamination levels based on that transfer. Note that in our model there is no direct pathogen transfer from an HCW to a patient or vice versa. This transfer pathway is indirectly considered by assuming that colonized patients shed pathogen and increase the contamination level of their rooms, and the contamination level of the room affects the probability of HCWs picking up pathogen from that room.

The submodel begins by determining the risk level of the task each HCW will perform at a particular time step. Type 1 HCWs have a greater chance of performing

low-risk tasks while Type 2 HCWs have a greater chance of performing high-risk tasks. The specific probabilities used are shown in Table 4. The risk level of the task is chosen at every 15-minute time step for both Type 1 HCWs and Type 2 HCWs. Therefore, a Type 2 HCW may perform different risk level tasks while with the same patient.

After determining the task-risk level, this submodel determines both the probability that an HCW will transfer pathogen to a room and the probability that a room will transfer to an HCW. Both of these probabilities depend on the risk level of the task being performed and on the amount of contamination already in the room or on the HCW's hands. We use three transfer functions, one for each risk level, to determine these probabilities. Each is a function of the following form, where x represents the contamination level (of the room or of the HCW) and k depends on the risk level of the task: The value of k controls the steepness of the curve. In particular, we use $k = 1, 0.5$, and 0.15 for a low-risk, medium-risk, and high-risk task, respectively. The resulting transfer functions are shown in Figure 1. The higher the contamination level of the HCW (or the room), the higher the probability of transfer. For a high-risk task, there is a greater chance of transfer at lower contamination levels than there is for a medium-risk or low-risk task at the same contamination level. Note that this process is only used for non-quarantined patients. For those patients who are quarantined, there is either a 100% chance of transfer or a 0% chance of transfer, depending on the variable *HCW-compliance-CDI-patient*. We set this value to 0.6 [5] since HCWs are more likely to comply with proper contact protocol when visiting a quarantined patient. When an HCW does comply with contact regulations with a quarantined patient, then there is a 0% chance of pathogen transfer; when an HCW does not, there is a 100% chance of transfer.

The final process in this submodel involves the actual transfer of pathogen. If is determined that an HCW will transfer pathogen to the room, then he or she will transfer 90% of his or her total contamination level to the room. This then decreases the HCW contamination level by 90% and increases the room contamination level by that same amount. This percentage is represented by the global variable *hcw-transfer-percent*. Similarly, if it is determined that a room will transfer pathogen to an HCW, then the room contamination level will decrease by 10%, and the HCW contamination level will increase by that amount. This value for *room-transfer-percent* was chosen with the assumption that, because a room has many surfaces on which pathogen may live, one HCW will only pick up a small percentage of the total contamination in one visit.

2.10 HCW compliance

Each time HCWs leave a room, they follow proper protocol with a 45% chance of effectively doing so [5]. Based on this probability, if it is determined they will effectively do so, they reduce their total contamination level to 10% of what it was prior to implementing protocol. If not, their contamination level remains the same.

2.11 Shift change and update of HCW time characteristics

Once an HCW's time since beginning a shift reaches the total length of his or her shift, assigned upon arrival (Section 2.1), he or she will leave the hospital. After

Type 1

Task Risk Level	Probability
low	0.60
medium	0.25
high	0.15

Type 2

Task Risk Level	Probability
low	0.15
medium	0.25
high	0.60

Table 4: Probabilities used to determine risk level of tasks performed by HCWs

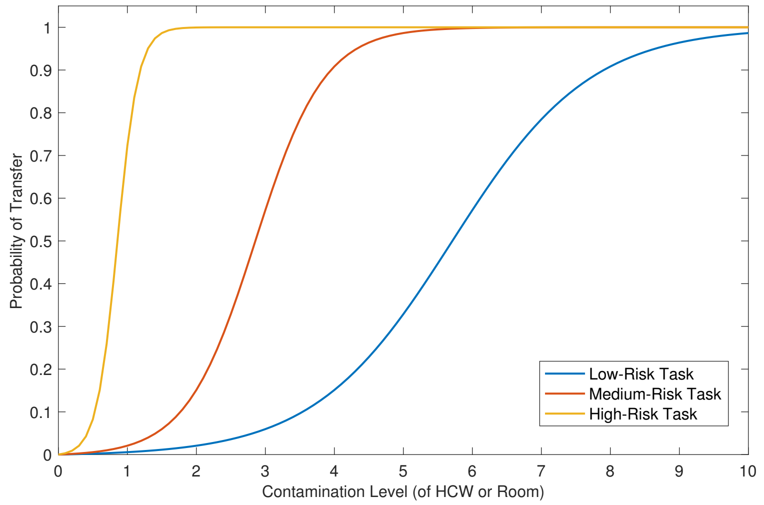

Figure 1: Transfer functions used to determine the probability a room (or HCW) will transfer pathogen at a particular time step

this, we update the counter tracking the time since each HCW's shift began before starting the next 15-minute time step.

2.12 Antibiotic assignment

The degree of microbiota disturbance (and resulting susceptibility to colonization) caused by antibiotics depends on the spectrum, duration, and number of antibiotics received [2, 16, 17]. For this reason, we maintain the three risk levels for antibiotics defined in [2]: low risk, high risk, and very high risk. This categorization of antibiotics was based on studies completed to analyze the association of particular antibiotics with *C. difficile*. In the antibiotic assignment submodel, we assign an antibiotic-risk level based on the probabilities of the antibiotic being low risk, high risk, or very high risk in terms of its association with *C. difficile*. The risk level of an antibiotic also affects the time until microbiota returns to normal, which we assign in this subroutine based on the risk level assigned. For all patients, we set the length of treatment to one week as a simplifying assumption also used in [2].

	Antibiotic risk level	Room contamination level	Value
p_l^h	low	high	0.0333
p_h^h	high	high	0.0920
p_{vh}^h	very high	high	0.1301
p_l^m	low	medium	0.0250
p_h^m	high	medium	0.0748
p_{vh}^m	very high	medium	0.1119
p_l^l	low	low	0.0167
p_h^l	high	low	0.0544
p_{vh}^l	very high	low	0.0874

Table 5: Probabilities of becoming colonized for each combination of antibiotic-risk and room-contamination level

2.13 Colonization probability assignment

At each half-day time step, a susceptible patient's probability of being exposed to *C. difficile* and becoming colonized depends on two things: the contamination level of the room and the risk level associated with the antibiotic received. To determine this probability, we divide room contamination levels into three categories: low, medium, or high contamination. Together with the three antibiotic-risk levels (low, high, or very high), this makes nine possible combinations of antibiotic-risk and room-contamination levels, which gives us nine different probabilities of colonization.

We denote p_h^l to represent the probability of a susceptible patient becoming colonized given that he or she is in a room with low contamination and has received a high-risk antibiotic. We use this notation similarly for the eight remaining probabilities, where the superscript refers to the room contamination level (l, m, or h) and the subscript refers to the risk level associated with the antibiotic (l, h, or vh).

To determine values for each of these probabilities, we began by using those calculated by Bintz et al. in [2]. Because studies have quantified the odds ratios for the risk of infection assigned to specific antibiotics [18] [19] [16], Bintz and his coauthors used odds ratios to represent the chances of becoming colonized if given a high-risk or very high-risk antibiotic compared to the odds of becoming colonized after given a low-risk antibiotic. Specifically, they were able to estimate odds ratios for high-risk and very high-risk antibiotics and then use those values, along with the data for the number of nosocomial infections, to determine the probabilities.

In our model, we used the nine probabilities calculated in [2] as a foundation but then modifed each of these values so that our number of nosocomial colonizations more closely matched the dataset. To make this match, we divided the nine probabilities used by Bintz et al. by the same scaling factor so that nosocomial colonizations accounted for 20% of all admissions [11, 12]. The final values we used for the probabilities are given in Table 5.

2.14 Resistance-restoration probability assignment

Once the microbiota returns to normal, a patient's resistance to colonization by *C. difficile* is restored. The chances of regaining resistance depend on how long a patient has been on an antibiotic and the type of antibiotic the patient received since

the associated antibiotic risk affects the length of time until a normal microbiota is restored.

To determine the probability that resistance will be restored for a patient at a particular time step, we use the same function Bintz et al. use in [2]. This function is logistic, where the input t represents the time since a patient began taking an antibiotic and T is the sum of the treatment length and the time until a normal microbiota is restored:

$$p(t,T) = \frac{1-p_{\min}}{1+\exp\left(-\frac{12}{T}\left(t-\frac{T}{2}\right)\right)} + p_{\min}.$$

The variable $p_{\min}$ is set to 0.2 and represents the minimum probability of regaining resistance while the parameter value of 12 controls the steepness of the curve.

2.15 Quarantine and treat

This submodel allows us to identify those patients who were successfully screened for CDI, mark them as quarantined, and model the effects of their isolation. Once patients are quarantined, they begin antimicrobial treatment for CDI, so we assign them an antibiotic using the procedure described in Section 2.12. Then, we update the patient's total number of hospital antibiotics to reflect this and initialize the time since beginning treatment to 0.

2.16 Patient length of stay

Depending on their disease status at admission, patients' length of stay in the hospital will vary. We assign patients a length of stay upon admission (Section 2.2) and determine this length in the same manner used in [2]. In their model, the authors resampled from the dataset generated by the Barnes-Jewish Hospital values for the length of stay of patients in each particular disease status. They then determined that resistant patients will stay between 0 and 16 days, susceptible and colonized patients will stay between 0 and 34 days, and diseased patients will stay between 0 and 80 days.

Author details

[1]Department of Engineering, Computing, and Mathematical Sciences, Lewis University, 1 University Parkway, 60446 Romeoville, IL,. [2]Department of Population Health and Pathobiology, North Carolina State University, 1052 William Moore Drive, 27606 Raleigh, NC,. [3]Department of Mathematics, University of Tennessee, 1403 Circle Drive, 37996 Knoxville, TN,. [4]Department of Electrical Engineering and Computer Science, University of Tennessee, 1520 Middle Drive, 37996 Knoxville, TN,. [5]School of Arts and Sciences, Johnson University, 37998 Knoxville, TN,. [6]Division of Infectious Disease, Washington University School of Medicine, 660 S Euclid Ave, 63110 St. Louis, MO,.

References

1. Railsback, S., Grimm, V.: Agent-based and Individual-based Modeling: a Practical Introduction. Princeton University Press, New Jersey (2012)
2. Bintz, J., Lenhart, S., Lanzas, C.: Antimicrobial stewardship and environmental decontamination for the control of *Clostridium difficile* transmission in healthcare settings. Bulletin of Mathematical Biology **79(1)**, 36–62 (2017)
3. Lanzas, C., Dubberke, E., Lu, Z., Reske, K., Grohn, Y.: Epidemiological model for *Clostridium difficile* transmission in healthcare settings. Infection Control and Hospital Epidemiology **32(6)**, 553–561 (2011)
4. Alasmari, F., Seiler, S., Hink, T., Burnham, C., Dubberke, E.: Prevalence and risk factors for asymptomatic *Clostridium difficile* carriage. Clinical Infectious Diseases **59(2)**, 216–222 (2014)
5. Rubin, M., Jones, M., Leecaster, M., Khader, K., Ray, W., Huttner, A., Huttner, B., Toth, D., Sablay, T., Borotkanics, R., Gerding, D., Samore, M.: A simulation-based assessment of strategies to control *Clostridium difficile* transmission and infection. PLoS ONE **8(11)**, 80671 (2013)
6. Gerding, D., Muto, C., Owens, R.: Measures to control and prevent *Clostridium difficile* infection. Clinical Infectious Diseases **46(Supplement 1)**, 43–49 (2008)

7. Leffler, D., Lamont, T.: *Clostridium difficile* infection. The New England Journal of Medicine **372(16)**, 1539–1548 (2015)
8. Dubberke, E., Haslam, D., Lanzas, C., Bobo, L., Burnham, C.-A., Grohn, Y.: The ecology and pathobiology of *Clostridium difficile* infections: An interdisciplinary challenge. Zoonoses Public Health **58(1)**, 4–20 (2011)
9. Wilcox, M., Mooney, L., Bendall, R., Settle, C., Fawley, W.: A case-control study of community-associated *Clostridium difficile* infection. Journal of Antimicrobial Chemotherapy **62**, 388–396 (2008)
10. Galvin, S., Dolan, A., Cahill, O., Daniels, S., Humphreys, H.: Microbial monitoring of the hospital environment: why and how? Journal of Hospital Infection **82**, 143–151 (2012)
11. Crobach, M., Vernon, J., Loo, V., Kong, L., Pèchinè, S., Wilcox, M., Kuijper, E.: Understanding *Clostridium difficile* colonization. Clinical Microbiology Reviews **31(2)**, 00021–17 (2018)
12. Kachrimanidou, M., Malisiovas, N.: *Clostridium difficile* infection: a comprehensive review. Critical Reviews in Microbiology **37**, 178–187 (2011)
13. Planche, T., Aghaizu, A., Holliman, R., Riley, P., Poloniecki, J., Breathnach, A., Krishna, S.: Diagnosis of *Clostridium difficile* infection by toxin detection kits: a systematic review. The Lancet Infectious Diseases **8(12)**, 777–784 (2008)
14. McFarland, L.: Update on the changing epidemiology of *Clostridium difficile*-associated disease. Nature Clinical Practice Gastroenterology and Hepatology **5(1)**, 40–48 (2008)
15. Furuya-Kanamori, L., J, M., Yakob, L., Riley, T., Paterson, D., Foster, N., Huber, C., Clements, A.: Asymptomatic *Clostridium difficile* colonization: epidemiology and clinical implications. BMC Infectious Diseases **15**, 516 (2015)
16. Slimings, C., Riley, T.: Antibiotics and hospital-acquired *Clostridium difficile* infection: update of systematic review and meta-analysis. Journal of Antimicrobial Chemotherapy **69(4)**, 881–891 (2014)
17. Dancer, S., Kirkpatrick, P., Corcoran, D., Christison, F., Farmer, D., Robertson, C.: Approaching zero: temporal effects of a restrictive antibiotic policy on hospital-acquired *Clostridium difficile*, extended spectrum β-lactamase-producing coliforms and methicillin-resistant *Staphylococcus aureus*. International Journal of Antimicrobial Agents **41(2)**, 137–142 (2013)
18. Bignardi, G.: Risk factors for *Clostridium difficile* infection. Journal of Hospital Infection **40(1)**, 1–15 (1998)
19. Feazel, L., Malhotra, A., Perencevich, E., Kaboli, P., Diekema, D., Schweizer, M.: Effect of antibiotic stewardship programmes on *Clostridium difficile* incidence: a systematic review and meta-analysis. Journal of Antimicrobial Chemotherapy **69(7)**, 1748–1754 (2014)
